# Unwanted hydrolysis or α/β-peptide bond formation: how long should the rate-limiting coupling step take?†

**DOI:** 10.1039/c9ra06124j

**Published:** 2019-09-27

**Authors:** Viktória Goldschmidt Gőz, Adrienn Nagy, Viktor Farkas, Ernő Keszei, András Perczel

**Affiliations:** MTA-ELTE Protein Modeling Research Group Pázmány P. stny. 1/A 1117 Budapest Hungary perczel.andras@ttk.elte.hu; Laboratory of Structural Chemistry and Biology, Institute of Chemistry, Eötvös Loránd University Pázmány P. stny. 1/A 1117 Budapest Hungary; Chemical Kinetics Laboratory, Institute of Chemistry, Eötvös Loránd University Pázmány P. stny. 1/A 1117 Budapest Hungary

## Abstract

Nowadays, in Solid Phase Peptide Synthesis (SPPS), being either manual, automated, continuous flow or microwave-assisted, the reaction with various coupling reagents takes place *via in situ* active ester formation. In this study, the formation and stability of these key active esters were investigated with time-resolved ^1^H NMR by using the common PyBOP/DIEA and HOBt/DIC coupling reagents for both α- and β-amino acids. Parallel to the amide bond formation, the hydrolysis of the α/β-active esters, a side reaction that is a considerable efficacy limiting factor, was studied. Based on the chemical nature/constitution of the active esters, three amino acid categories were determined: (i) the rapidly hydrolyzing ones (*t* < 6 h) with smaller (Ala) or even longer side chains (Arg) holding a large protecting group; (ii) branched amino acids (Ile, Thr) with slowly hydrolyzing (6 < *t* < 24 h) propensities, and (iii) non-hydrolyzing ones, such as the hard-to-couple β-amino acids or β-sugar amino acid derivatives, stable for longer times (*t* > 24 h) in solution. The current insight into the kinetics of this key hydrolysis side reaction serves as a guide to optimize the coupling conditions of α- and β-amino acids, thereby saving time and minimizing the amounts of reagents and amino acids to be used – all key factors of more environmentally friendly chemistry.

## Abstract 2

Parallel to the amide bond formation, the hydrolysis of the active esters of α/β-amino acids, as an unwanted side reaction limiting coupling efficacy, is studied.

## Graphical abstract

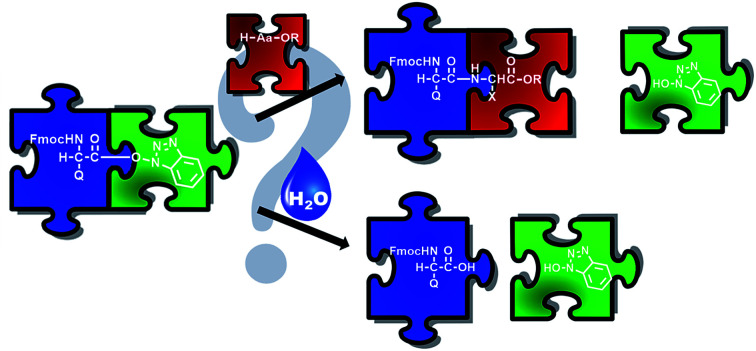

## Introduction

Both polypeptides and proteins are evolutionarily fine-tuned linear polymers, playing key roles in almost every process of cellular life.^1^ The need for polypeptide-based lead-compounds and drugs (*e.g.* oxytocin, insulin, GLP1 agonists) is increasing, enhancing the role of polypeptides in drug discovery.^2–5^ Thus, the efficient synthesis of oligo- and polypeptides has been a current topic and a continuous challenge of synthetic chemistry since the second half of the last century. Solid phase peptide synthesis (SPPS) became a technique developed and fine-tuned ever since.^6^ Both the chemistry of introducing linkers, protecting groups, coupling agents, resins, *etc*. and the engineering at a given level of automation (microwave-assisted, continuous-flow, *etc*.) have been improved.^7–15^

A generalized, highly automated and optimized method for amide bond formation for peptide synthesis is now well established: the activation of the carboxyl group *via in situ* active ester formation is typical. As a result of a large number of coupling agents probed and different kind of active esters tested,^16,17^ perhaps the most commonly used pair of reagent today is that of the HOBt/DIC, introduced 20 years ago.^7^ In addition, for coupling “difficult sequences” either PyBOP/DIEA or HATU/DIEA reagents were developed and used the most often in combination with the basic HOBt/DIC^18,19^ protocol. One of the additional advantages of using PyBOP is to minimize or even avoid racemization of the coupled amino acid residue. In contrast to this, when using HATU the chance of racemization increases with the length of the coupling time and thus, gives a time limit on the overall reaction, which should be set shorter than 3 h.^20^ According to the standard coupling protocols worked out for α-amino acid residues, amino acids are pre-activated with the coupling agent before mixing with the (*i* − 1) amino acid (R–NH_2_) which requests about 10 minutes in general.^21^ The typical coupling time of the residue (*i* − 1) to the *i* varies between 1 to 3 hours for α-amino acids, occasionally increased up to 18 hours for a difficult to couple residue.^22^ However, these “common” time limits rely on thumb-rules and common understanding of peptide chemists, in default of the support of quantitative and systematic kinetic studies. Some active esters are commercially available, but usually it is made *in situ* during the coupling step. Active esters are generally investigated in details only when they are “first described” as a new coupling agent, like in the case of PyBOP for example, when the formation of the active esters was followed by TLC.^20^ In most cases the time required for their formation was determined and the mechanism of activation and coupling was examined.^16^ After the 1990s, each of the new coupling reagents was tested and compared to the others with respect to make peptides according to the literature data (*e.g.* ACP),^23^ however the comprehensive analysis of the nascent active ester's stability was not checked and probed. Thus, studies on the formation and stability of amino acid active esters in the literature are sporadic, rare and often quite inconsistent. In the seminal work of Albericio and co-workers, the stability of some active esters in DMF was followed by HPLC.^7^ However, the active esters of the most common α- and β-amino acid residues have not yet been systematically tested. The dry DMF typically used for coupling contains a little, but a significant amount of water (*e.g.* ≤0.03% according to Sigma Aldrich). Furthermore, an inert atmosphere is sometimes used during residue coupling, thus these traces of water molecules deeply perturbing the reaction, as besides the peptide bond formation of interest, the hydrolysis of the water sensitive active esters proceeds as a side reaction. Recently, we have tested some commonly used coupling reagents for selected Fmoc-protected β-sugar amino acids, Fmoc-β-SAA-OH,^24,25^ indeed hard to activate and couple.^26^ Considering the time needed for the active ester to be formed without hydrolysis for SAA derivatives the PyBOP/DIEA system turned out to be the best choice with respect to coupling efficacy, in parallel to minimize or totally avoid racemization.

Here we present a systematic study on the kinetic details of both making and preserving active esters from an array of both linear and cyclic, α- and β-amino acid residues with PyBOP and HOBt. As some especially β-amino acid and sugar amino acid residues are of elevated costs and sluggish to couple, the optimization of these reaction conditions is far to be a luxury. Our goal was to establish a general relationship between the chemical nature and the molecular topology of these amino acids with respect to their ability of coupling. Most importantly, the hydrolysis of their active esters was in the focus, as their stability in the presence of some water seems to be the key step and the limiting factor that influences and limits both the coupling time and efficacy. In addition, the molecular excess of the amino acid needed to achieve a total coupling efficiency (*e.g.* >98%) was also a key factor to be determined. If we could determine and categorize the activity and stability of the active esters of the amino acids of different molecular topology, then peptide synthesis will be conducted in a more time- and cost-effective way. Moreover, with these data at hand, the time requirement of the automated protocols of pre-activation and coupling could be fine-tuned and optimized at a partner specific manner.

## Results and discussion

**Scheme 1:**
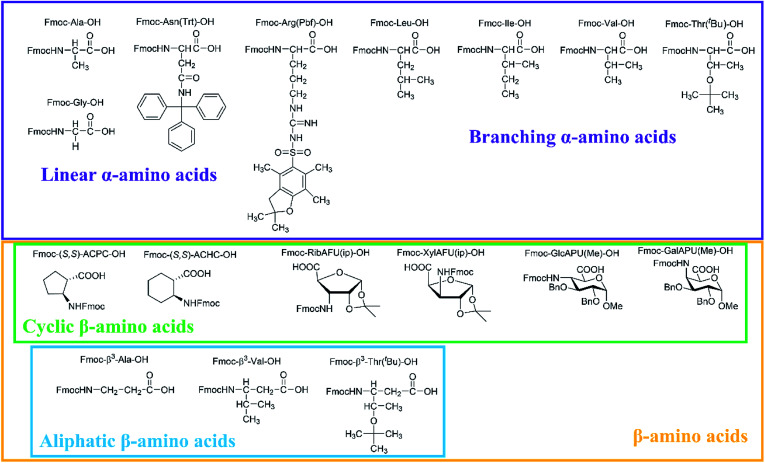
Three different groups of the α- and β-amino acids studied: (i) α-amino acids (unbranched residues) have either shorter or longer side chains with or without protecting groups, (ii) residues with β- and/or γ-branched side chains and (iii) aliphatic and cyclic residues of a rigid structure equipped of large protecting groups.

In our comprehensive analysis, the use of PyBOP/DIEA coupling reagent pair was studied with amino acid residues of different chemical constitution. These selected amino acid residues represent most properties of common proteinogenic amino acids. They have similarly polar or apolar character, they are either protected or un-protected and have a constitution comprising a small or a large side chain in fact either easy- or hard-to-couple to the adjacent amino acid residues. α-Amino acids having either no/shorter –H (Gly), –CH_3_ (Ala) or longer side chains –CH_2_–CONH– (Asn) and –(CH_2_)_3_NHC(NH)NH– (Arg) with large protecting groups (Trt, Pbf, respectively) form the first group. α-Amino acids with β- and/or γ-branched side chains, such as Thr, Leu, Ile, and Val constitute the second group. Aliphatic or cyclic β-amino acids (normal and β-sugar amino acids) with a rigid structure and large protection form the third group. The latter two comprise the so-called hard-to-couple residues as well (Scheme 1).

**Fig. 1:**
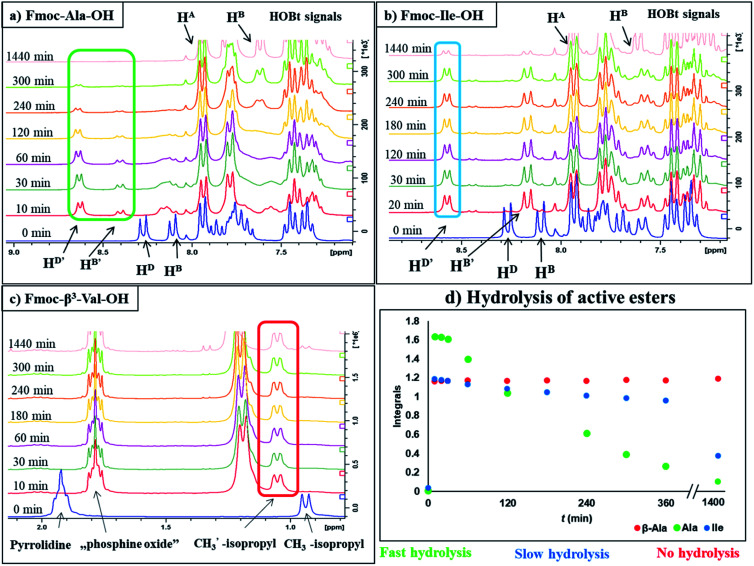
^1^H NMR resonance frequency and intensity changes as a function of the time (*T* = 25 °C) during the active ester formation and hydrolysis of the three different groups of amino acids. Selected ^1^H-resonances (*e.g.* H^D′^, H^B′^ as the aromatic ring protons of active ester or H^ip′^ as the side chain protons of active ester) are used to monitor and analyze the kinetics of the reactions. (a) NMR spectra of the quickly hydrolyzed active ester from Fmoc-Ala-OH with PyBOP, (b) NMR spectra of the slowly hydrolyzed active ester from Fmoc-Ile-OH with PyBOP, (c) NMR spectra of the stable active ester from Fmoc-β^3^-Val-OH with PyBOP, (d) the integral–time diagram of three different group of amino acids referenced to the signal of DMF-d_7_: residue of fast hydrolyzing property is reported in green, while slow hydrolysis is depicted with blue and non-hydrolyzing residue with red.

To optimize the coupling of an amino acid in a specific manner both the active ester formation and its stability were followed by time-resolved ^1^H NMR spectroscopy. The solution of the amino acid was mixed with PyBOP or HOBt in an equimolar amount in DMF-d_7_ at 25 °C. As expected, the PyBOP or HOBt and the N-protected amino acid form a stable and unreactive mixture for 24 hours (stability checked by NMR). At *t* = 0 DIEA (in the case of PyBOP) or DIC (in the case of HOBt) was added to initiate the active ester formation. ^1^H NMR spectra were recorded after 10, 20, 30 and 60 minutes followed by hourly acquisition in the first 6 hours, and finally after 24 hours. (Note, that in SPPS the typical coupling time is 1 or 3 hours). To perform a quantitative analysis first, the non-overlapping, characteristic resonance frequencies were assigned and monitored. Usually, the integrals of the selected aromatic protons of the active esters (*e.g*. for Fmoc-Ile-OH, H^D′^ at 8.57 ppm or H^B′^ at 8.16 ppm, Fig. 1b) or characteristic side chain protons (*e.g*. isopropyl group for Fmoc-Val-OH at 1.05 ppm, Fig. 1c, or H^α^ between 4.4–5.5 ppm in ESI†) were considered.

The active ester is formed within 10 minutes for all α-, except a few β-amino acids, which requested longer reaction times (*e.g.* Fmoc-β^3^-Thr(^*t*^Bu)-OH: 20 min, Fmoc-ACPC-OH: 540 min). However, even for these slowly reacting residues, their active esters are formed in a ratio of more than 50% after 10 minutes: a ratio in principle sufficient to achieve a successful coupling (The last column of Table 1 shows the conversion of formation or hydrolysis of an active ester after 10, 60 and 180 min). Based on this data the typical coupling time can be determined for each amino acid with PyBOP. For example, the active ester of Fmoc-Arg(Pbf)-OH hydrolyzed quickly (conversion of hydrolysis is 97% after 3 hours, Table 1). Therefore, it seems enough to couple such an active ester only for a single hour, and unnecessary to continue the same reaction for additional hours. In contrast, β-amino acids can be coupled for a longer time (*e.g.* 18 hours) as their active esters were found to be stable at least for 24 hours.

**Table 1:** Reaction times (min) and conversions (%) of the active ester formation (f) and hydrolysis (h) for the Fmoc protected α- and β-amino acids of the three molecular topology groups. Conversion after 10, 60 minutes and 3 hours^a^ is shown which indicates the limit of the coupling

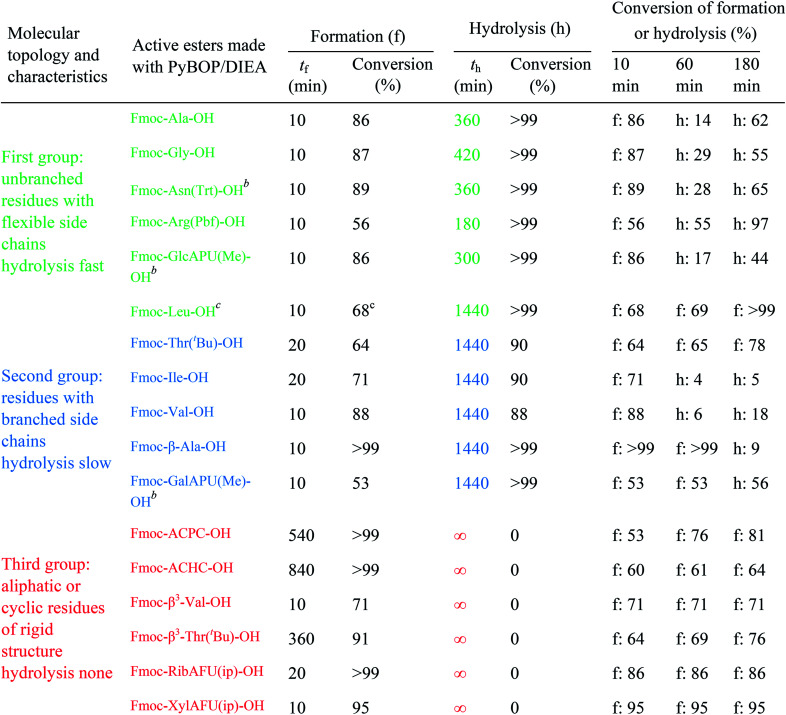

a 10 min: typical time of active ester formation; 1 and 3 hours: typical reaction time used for coupling.

b Active ester was decomposed.

c Active ester is formed >99% conversion after 3 hours.

**Scheme 2:**
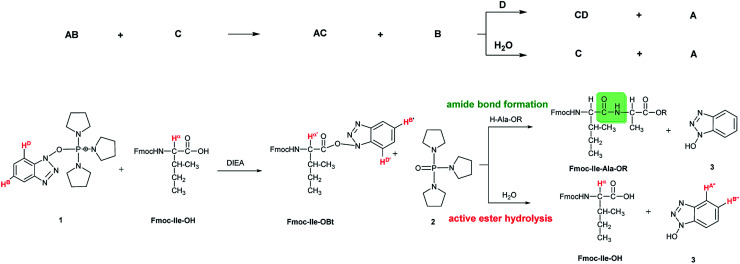
The formation and hydrolysis of the active ester made from α-amino acid (Fmoc-Ile-OH) with PyBOP/DIEA coupling reagent pair. AB = PyBOP (1); C = amino acid; AC = active ester; B = phosphine oxide (2), D = base; CD = unidentified by-product, A = HOBt (3). The ^1^H resonance frequencies of the atoms highlighted with red (H^D^, H^B^, H^α^, H^α′^, H^D′^, H^B′^, H^A′′^, and H^B′′^) were used to monitor the reaction. Their changes in intensity as a function of time were recorded and used to construct suitable kinetic models.

In some cases, due to the presence of the residual water, hydrolysis of the active esters was found to be quite substantial and thus, the starting amino acids were recovered completely after 24 hours (Scheme 2 and Table 1). However, when the mixture of the solvent (DMF-d_7_) and HOBt was dried on molecular sieves to remove the water content of HOBt·H_2_O, no formation of active esters was observed. We do explain this phenomenon, as most probably the absence of water destabilizes HOBt and thus, the lack of sufficient HOBt resulted in the failure of the coupling reaction. Thus, a minimum amount of water is requested for active ester formation. According to the manufacturers, the water content of the dedicated NMR solvents (*e.g.* DMF-d_7_, ≤0.05%, data from Eurisotop: 0.045–0.02%) is practically the same as that used during peptide synthesis (≤0.03% in DMF according to Carlo Erba 0.03–0.015%). Moreover, the actual H_2_O content of the solvents based on the calculations using the kinetic model was found higher in most cases, [H_2_O]_calc_ = 0.02–0.25 mM, than the value indicated by the supplier [H_2_O]_origin_ = 0.016–0.027 mM confirming that DMF indeed contains a small, but significant amount of water. (This latter concentration was calculated based on 0.03–0.05% of water content in 550 μl DMF-d_7_ in the NMR tube). During the current measurements, several stocks of dried DMF-d_7_ were probed, 2–3 parallel experiments were completed for each. Additional evidence of the ongoing hydrolysis was the appearance of HOBt (3) (*e.g.* signal at 7.62 ppm, H^A′′^, Fig. 1), as a by-product, identified by NMR measurements. In conclusion, if using the conventionally accepted ‘blind’ methodology and types of solvents, then the hydrolysis of the *in situ* active ester indeed proceeds, and thus, active ester formation and hydrolysis are two competing parallel reactions. Therefore, it is important to focus and understand this side reaction, in order to conduct the main reaction properly, the amide bond formation as effectively as possible. By measuring and working out the kinetics of these coupled reactions, the optimum time and conditions for coupling of α- and β-amino acids can now be determined with confidence. Thus, both saving time and minimizing the extent of reagents and amino acid derivatives necessary to use become possible.

For the amino acids of the above three groups, we have established quantitative differences in terms of their rate of hydrolysis. Data retrieved from the ^1^H-integrals of selected not overlapping resonances as a function of the time (Fig. 1d) are different. For the integral–time diagram the values were referenced to DMF-d_7_: signal at 2.75/2.93 ppm was considered as unity (integral = 1.0). In the case of “fast hydrolysis”, we found that the active ester decomposes within 6 h (*t* < 360 min) at a conversion > 90% (Fig. 1a and d/green points) and the starting Fmoc-AA-OH is regained quite substantially. For “slowly hydrolyzing” active esters, a similar amount of decomposition takes place between 6–24 h (1440 min: Fig. 1b and d/blue points), while the active esters of Fmoc-AA-OHs of the third group remain stable: their decomposition is still limited (<5%) even after 24 h (Fig. 1c and d/red points) and might be called as “non-hydrolyzing” residues.

**Fig. 2:**
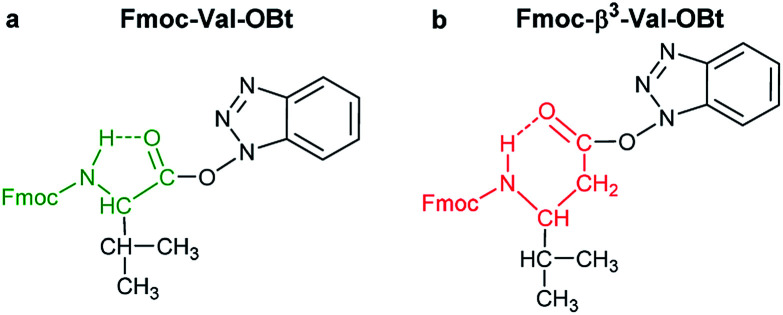
The stabilizing H-bond in an active ester: (a) 5-membered H-bonded pseudo-ring is formed in case of α-amino acids (highlighted with green), whereas (b) an even more stable 6-membered H-bonded pseudo ring emerges in β-amino acids (highlighted with red)

(i) A fast hydrolyzing amino acid has typically either no/short side chain (*e.g.* Gly, Ala) or a long one (*e.g.* Arg, Asn) equipped even with a larger protecting group (Trt, Pbf). However, if these masked functional groups are sufficiently far from the reaction center (C′=O) and thus they do not interfere with the backbone atoms, then they cannot act against hydrolysis. The only exception was Fmoc-Leu-OH, which – unlike all the amino acids – did not form the active ester within 10 minutes (Table 1). Its maximum conversion was only 68% and did not change until 3 hours, but after that, it was >99% and started to hydrolyze quickly. This is the reason why it appears in the first group, despite the fact that it has a γ-branch. (ii) If an amino acid has either a β- or a γ-branch, the hydrolysis proceeds slower due to the steric hindrance of the side chain on the C′=O. In some cases, the active esters were stable for 1–3 hours, *e.g*. Fmoc-Thr(^*t*^Bu)-OBt started to hydrolyze after 3 hours. (iii) Active esters formed from β-amino acids are stable within the time frame of the current observation. The 6-membered H-bond pseudo ring structure gives higher stability compared to that of 5-membered present in α-amino acid active esters (Fig. 2). (iv) In the case of β-sugar amino acids, the flexibility of the pyranoside ring^27^ causes the hydrolysis of Fmoc-GlcAPU(Me)-OH and Fmoc-GalAPU(Me)-OH (Table 1). Due to the *cis* and *trans* configurations, the hydrolysis was slower for the d-*galacto* configuration (1440 min, Table 1) than for the d-*gluco* derivative (300 min, Table 1) and thus it belongs to the second group due to its characteristics regarding hydrolysis. While, those of the furanoid ring (β-SAAs: Fmoc-RibAFU(ip)-OH and Fmoc-XylAFU(ip)-OH) are indeed no hydrolyzing, because they have bicyclic, rigid structures which prevent hydrolysis. (v) Slow hydrolysis was observed for Fmoc-β-Ala-OH as the increased backbone flexibility presents some obstacle against a fast hydrolysis.

### Kinetic analysis

To determine kinetic parameters as *e.g.* the half-life and starting concentration of Fmoc-AA-OH active esters, a kinetic analysis of the temporal evolution of the reactions was performed. The hydrolysis of the active esters proved to be a second-order reaction. This was also supported by the fact that the measured ([AC]_0,meas_) and calculated ([AC]_0,calc_) initial concentration of the active esters were in good agreement. However, for some active esters, [AC]_0,calc_ was significantly larger than [AC]_0,meas_; in such cases, the hydrolysis started before acquiring the first measured point (*i.e*. 10 min). This behavior occurred in the case of amino acids which hydrolyze fast (group one). From the kinetic analysis, the initial concentration of water ([H_2_O]_0_) could also be estimated, except for a few cases where the uncertainty of this parameter was very large, due to the lack of enough experimental points. In these cases, the initial concentration of water has been fixed to an average of the estimated water content of DMF (0.25 mM) found in reactions where this parameter could be determined at a small uncertainty.

As the hydrolysis follows second-order kinetics, the half-life (*t*_1/2_) of active esters depends on the initial concentration of water in the reaction mixture as follows:1
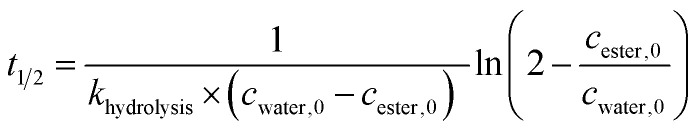
where *k*_hydrolysis_ is the rate constant of the hydrolysis, *c*_water,0_ is the initial concentration of water, and *c*_ester,0_ that of the active ester. (Note that this formula is valid only if *c*_water,0_ is greater than *c*_ester,0_ – as is the current case. If *c*_ester,0_ exceeds *c*_water,0_ but it is not higher than twice the value of *c*_ester,0_, then the two initial concentrations should be flipped in both the difference and the fraction. If *c*_ester,0_ exceeds *c*_water,0_ by more than a factor of 2, then the active ester concentration cannot become as low as half of the initial concentration, due to the hydrolysis.)

The half-lives were determined for the estimated initial ([H_2_O]_0_) and for fixed water concentration ([H_2_O]_0_ = 0.25 mM) also. For the latter, the [AC]_0_ was also fixed at 0.1 mM in order that the half-lives were more comparable. According to this, Fmoc-Leu-OH really belongs to the fast hydrolyzing amino acids, based on *k*_hydrolysis_ and *t*_1/2_ values. Nevertheless, it is interesting that Fmoc-Thr(^*t*^Bu)-OH and Fmoc-Val-OH would belong to this first group (Table 2). It means that, if there would be enough water in the solution, they would hydrolyze quickly (*t*_1/2,calc_ and *k*_hydrolysis_) according to the calculation. However, repeated measurements have always resulted in slow hydrolysis; so it is due to the steric hindrance of β-branch in the side chain, not the amount of water. For this reason, the half-life is less indicative concerning the rate of the hydrolysis; the correct comparison of the rates can be made based on the rate constant *k*_hydrolysis_ of the second-order reaction. Taking this into account, the grouping of active esters was independent of the actual water concentration; therefore, large differences in the rate of the hydrolysis were indeed caused by structural differences.

**Table 2:** Kinetic parameters (*k*_hydrolysis_, initial concentrations: [AC]_0_ and [H_2_O]_0_, half-lives: *t*_1/2,calc_) of the hydrolysis of active esters for group one and two. Errors after the ± signs are given as half widths of 95% confidence intervals of the parameters determined from the estimation

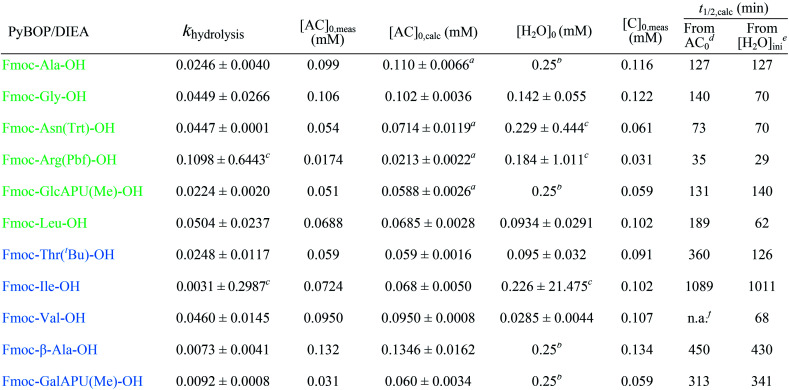

a Hydrolysis already started before the first measured point since [AC]_0,calc_ > [AC]_0,meas_.

b [H_2_O]_0_ was fixed at 0.25 mM during parameter estimation.

c The value not significant, confidence intervals are to large.

d [H_2_O] used as initial water concentration.

e With [AC]_0_ = 0.1 and [H_2_O] = 0.25 mM.

f Not applicable; the estimated water concentration was inferior to that of half of the active ester concentration.

Finally, the active ester formation with HOBt/DIC of selected amino acids of all the groups was also measured and compared with values obtained for PyBOP. The active esters are the same benzotriazole derivatives in both cases, but the mechanism of formation is different for HOBt/DIC and PyBOP/DIEA.^26,28^ Accordingly, it was not surprising that for all residues, the active esters were formed slower (60–240 min, Table 1, ESI†); however, the maximum conversions were also significantly smaller (35–73%). Nevertheless, this conversion is enough for coupling if 3 equivalent is used. Interestingly, the members of each group behaved in the opposite way than with the former coupling agents: (i) the slowly hydrolyzing Fmoc-Val-OBt was formed within 240 minutes but found stable for a long time (24 h). (ii) On the contrary, the previously stable Fmoc-RibAFU(ip)-OBt made from PyBOP turned out to hydrolyze faster to result in the starting β-SAA just after 1 hour! (iii) The fast hydrolyzing Fmoc-GlcAPU(Me)-OH was found also stable for 24 hours just like the Val residue. It means that if an active ester forms slowly, the formation and hydrolysis will be the two competitive reactions, not the amide bond formation and hydrolysis, therefore after the water runs out from the system the active ester becomes stable.

### Practical considerations

(i) Using HOBt/DIC as the coupling reagent pair, reaction time should be longer (2–3 h) then typical protocols propose, especially, if equimolar or only small molar excess of amino acid derivatives is used.

(ii) Pre-activation should be considered, from 10 min up to 1 hour, to avoid an incomplete coupling and thus, sequence errors to emerge, more prevalent perhaps for Arg. This was tested for shorter peptides (*e.g.* EEEAVRLYIQWLK) with continuous flow SPPS.^29^ We found for example, that without pre-activation of these amino acids, deletion of Arg and Asp was observed. Note that both belong to the first group based on their molecular topology.

(iii) Our current data support the use of PyBOP/DIEA, as faster and more complete coupling reactions were monitored for all types of residues. The use of PyBOP/DIEA is especially recommended for non-commercial, often inaccessible and thus expensive amino acids (special protected α-amino acids: *e.g*. Fmoc-Lys(Dde)-OH; β-amino acids or sugar amino acids).

(iv) For fast hydrolyzing amino acid residues (elements of the first group) a short coupling time (*t* < 1 hour) is recommended to be implemented. Using pre-activation (continuous flow SPPS protocol) worked out for small peptides (*e.g.* IFDPETGTWI),^29^ couplings were unsuccessful due to the poor stability of the active esters.

**Fig. 3:**
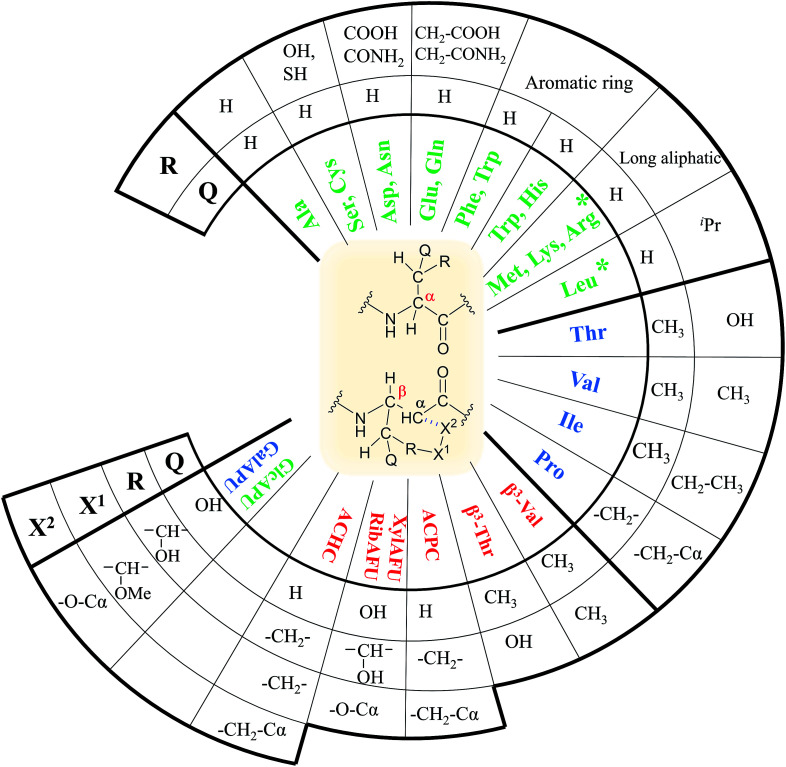
Classification of the proteinogenic α- and selected β-amino acids based on their molecular topology. Fast hydrolyzing ones are highlighted green, slow hydrolyzing ones with blue and no-hydrolyzing ones with red.

(v) In the case of difficult peptide sequences, after 3 hours of coupling time, we do recommend to complete a second coupling cycle, especially for expensive amino acids, by adding additional PyBOP to the coupling mixture directly. This was tested for the sequence –SGXGD– (X: -RibAFU(ip)-). After 3 hours of coupling time, with additional 3 molar equivalents of PyBOP the coupling efficacy has increased by 20%.

(vi) Slowly hydrolyzing amino acids (residues of the second group) are recommended to be coupled for longer time: *e.g.* 6 < *t*_coupling_ < 18 hours (overnight coupling). Adding (optional) surplus of PyBOP/DIEA to the coupling mixture after 6 hours could be advantageous.

(vii) A given excess of the coupling reagent and the amino acid will typically compensate the consequences of the hydrolysis and assure coupling. This was shown by the good coupling efficacy of Fmoc-RibAFU(ip)-OH with HOBt/DIC in a –GXXG– short chimera peptide by using 3 equivalent of sugar amino acid^26^ (despite of the fact, that this active ester is unstable). However, in the case of expensive amino acids (*e.g.* β-amino acids, special protected α-amino acids) it may be worth to reduce the excess of amino acids or to use other coupling reagents (*e.g.* PyAOP) or solvent (*e.g.* NMP).

(viii) Proteinogenic amino acids are thus proposed to couple as follows:

- in the case of Gly, Ala, Asn, Phe, Tyr, Cys, Met, Gln, Asp, Glu, Arg and Lys (members of the first group) coupling should be conducted as described in point iv.

- amino acids as Val, Pro, Ser, Thr, Leu, Trp, His and Ile (members of the second group) a successful coupling requires pre-activation or longer coupling time (3 h).

- finally, β-amino acid and β-sugar amino acid residues constitute the third group, where a much longer coupling time of 3–18 hours is needed (Fig. 3).

## Conclusion

A very important accompanying side reaction of peptide bond formation is quantitatively analyzed here. We have determined based on the formation and stability of the active esters of various α- and β-amino acids with PyBOP/DIEA or HOBt/DIC key kinetic parameters, by using time-resolved ^1^H NMR spectroscopy. It turned out that these species are able to hydrolyze parallel to the amide bond formation due to traces of water inherently present. Based on the kinetic data established for hydrolysis, three different groups of amino acid residues were identified. Amino acids having a small (*e.g.* G, A) or long side chain and equipped with a large protecting group (Asn, Arg), but placed far from the reaction center hydrolyzes quickly: within less than 6 hours. This fact indicates that it is necessary to couple these amino acids within a short range of time (*e.g.* <1 h). Active esters of amino acids having a β- and/or γ-branch in the side chain (*e.g.* Leu, Thr) hydrolyze much slower, between from 6 to 24 hours, and therefore – if needed – they can be coupled for a longer time (6–18 h) to achieve a more complete amide bond formation. Nevertheless, some amino acids, usually β-, were found to be resistant against hydrolysis for 24 hours or longer (*e.g.* ACPC, Fmoc-RibAFU(ip)-OH). Fortunately, these are typically hard-to-couple amino acids, thus they can be coupled for a longer time than the standard coupling protocol prescribes; even overnight coupling could be set up, as their active esters are stable enough to resist during such an extension of the reaction time. The high stability of β-amino acids comes from the six-membered H-bond pseudo-ring, which hinders both hydrolysis and coupling. To give a quantitative account of the true nature of the hydrolysis, a kinetic study was performed to estimate both the initial active ester and water concentrations, along with the rate constant of the hydrolysis. The results indicate that the different hydrolysis capacity of the amino acids does not depend on the concentration of water; it is rather caused by their diverse structure.

## Experimental section

Analytical data for all compounds: ^1^H NMR spectra and figures of active ester formation of all amino acids can be found in ESI,† in the online version.

### Reagents and instrumentations

Reagents, materials and solvents were obtained from Sigma Aldrich, Irish Biotech GMBH or Eurisotop. β-Sugar amino acids were synthetized in our laboratory based on our previous works.^24,25^

### NMR measurements

^1^H NMR experiments were performed at 298–300 K on Bruker Avance DRX 250 MHz spectrometer equipped with 5 mm SB dual probe with *z*-gradient, operating at 250.13 MHz for ^1^H and/or on a Bruker Avance III 700 spectrometer operating at 700.05 MHz using a Prodigy TCI H&F-C/N-D, *z*-gradient probe head. Spectra were recorded in DMF-d_7_ using the solvent residual peaks as the ^1^H internal reference: 2.75, 2.93 and 8.03 ppm. The sample concentrations ranged from 10 to 20 mM (Table 2). Spectra evaluation was completed within TopSpin 3.5 software.

### Kinetic analysis

Kinetic parameter estimation was based on the integral of selective NMR signals considered to be proportional to the concentration of the relevant species. The mechanism taken into account is the one shown on top of Scheme 2, but only the hydrolysis step was modeled. For the parameter estimation, the COPASI 4.16 (Build 104) Biochemical System Simulator software (http://copasi.org/) was used, with the parameter estimation option of the Levenberg–Marquardt method. The result of the estimation procedure did not depend on the choice of the initial parameters within a large interval, thus there was one stable optimum for the fit of the model only. Confidence interval half-widths were calculated from the estimated standard deviations based on the Student distribution with *n* − *p* degrees of freedom, where *n* is the number of data in the concentration *vs.* time measurements and *p* is the number of parameters estimated. Usually, three parameters were estimated: the rate constant of the hydrolysis *k*_hydrolysis_ and the initial concentrations of the active ester [AC]_0_ and that of the water [H_2_O]_0_. In some cases, the estimated water concentration was not significant; in these cases, we have fixed it to 0.25 mM, according to roughly the average of the estimated concentration in other cases.

## Compliance with ethical standards

This article does not contain any studies with human participants or animals performed by any of the authors.

## Conflicts of interest

The authors declare that they have no conflict of interest.

## Abbreviations

Fmoc-GlcAPU(Me)-OH: Methyl 2,3-di-*O*-benzyl-*N*-(9-fluorenylmethoxy-carbonyl)-4-amino-4-deoxy-α-d-glucopyranoside uronic acid
Fmoc-GalAPU(Me)-OH: Methyl 2,3-di-*O*-benzyl-*N*-(9-fluorenylmethoxy-carbonyl)-4-amino-4-deoxy-α-d-galactopyranoside uronic acid
Fmoc-RibAFU(ip)-OH: 1,2-*O*-isopropylidene-*N*-(9-fluorenylmethoxy-carbonyl)-3-amino-3-deoxy-α-d-ribofuranuronic acid
Fmoc-XylAFU(ip)-OH: 1,2-*O*-isopropylidene-*N*-(9-fluorenylmethoxy-carbonyl)-3-amino-3-deoxy-α-d-xylofuranuronic acid
Fmoc-ACPC-OH: *N*-(9-fluorenylmethoxy-carbonyl)-2-amino-cyclopentanecarboxylic acid
Fmoc-ACHC-OH: *N*-(9-fluorenylmethoxy-carbonyl)-2-amino-cyclohexanecarboxylic acid
Fmoc-AA-OH: Fmoc protected α-amino acid

## Supplementary Material

RA-009-C9RA06124J-s001

RA-009-C9RA06124J-s002

RA-009-C9RA06124J-s003

RA-009-C9RA06124J-s004

RA-009-C9RA06124J-s005

RA-009-C9RA06124J-s006

RA-009-C9RA06124J-s007

RA-009-C9RA06124J-s008

RA-009-C9RA06124J-s009

RA-009-C9RA06124J-s010

RA-009-C9RA06124J-s011

RA-009-C9RA06124J-s012

RA-009-C9RA06124J-s013

RA-009-C9RA06124J-s014

RA-009-C9RA06124J-s015

RA-009-C9RA06124J-s016

RA-009-C9RA06124J-s017

RA-009-C9RA06124J-s018

RA-009-C9RA06124J-s019

RA-009-C9RA06124J-s020

RA-009-C9RA06124J-s021

RA-009-C9RA06124J-s022

RA-009-C9RA06124J-s023

RA-009-C9RA06124J-s024

RA-009-C9RA06124J-s025

RA-009-C9RA06124J-s026

RA-009-C9RA06124J-s027

RA-009-C9RA06124J-s028

RA-009-C9RA06124J-s029

RA-009-C9RA06124J-s030

RA-009-C9RA06124J-s031

RA-009-C9RA06124J-s032

RA-009-C9RA06124J-s033

RA-009-C9RA06124J-s034
