# Supplementary figures and images for: Unwanted hydrolysis or α/β-peptide bond formation: how long should the rate-limiting coupling step take?
Source: RSC Adv. 2019 Sep 27;9(53):30720–8. doi: 10.1039/c9ra06124j (PMC9072530; doi:10.1039/c9ra06124j)

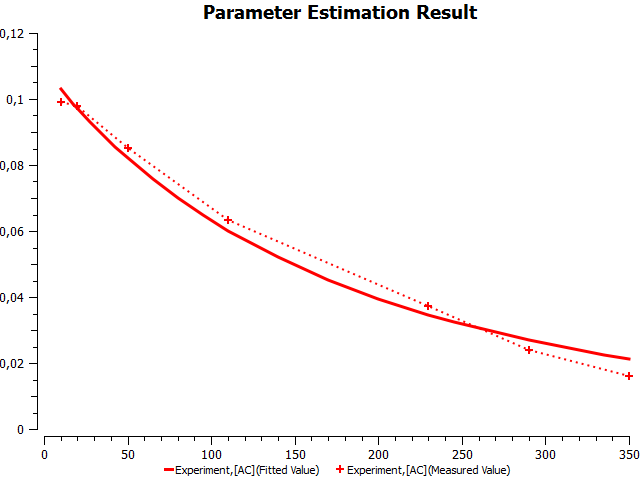

Supplement: RA-009-C9RA06124J-s001 [file RA-009-C9RA06124J-s001.tiff]

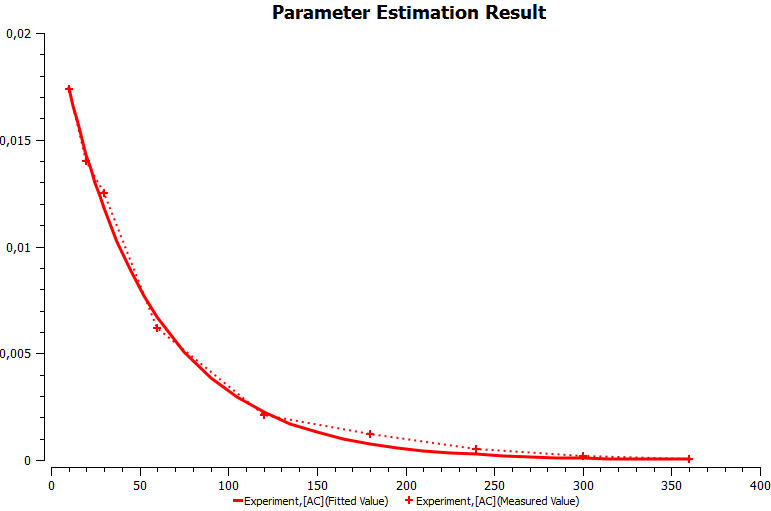

Supplement: RA-009-C9RA06124J-s004 [file RA-009-C9RA06124J-s004.tiff]

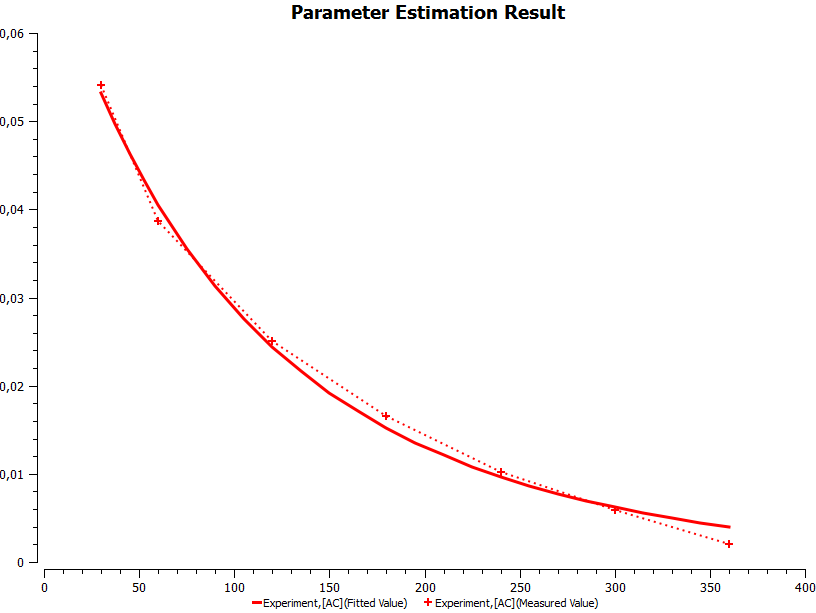

Supplement: RA-009-C9RA06124J-s007 [file RA-009-C9RA06124J-s007.tiff]

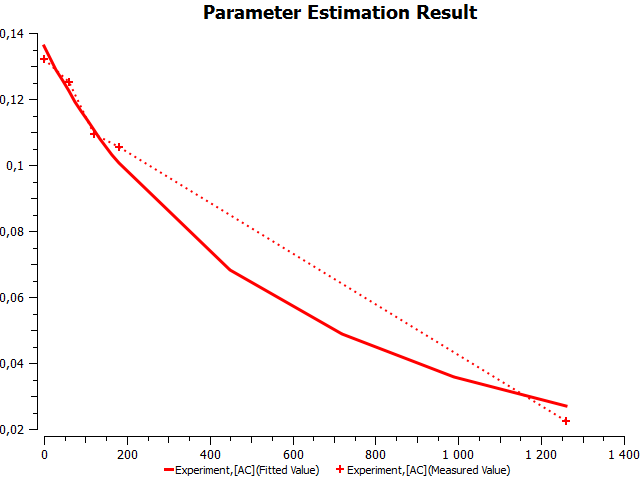

Supplement: RA-009-C9RA06124J-s010 [file RA-009-C9RA06124J-s010.tiff]

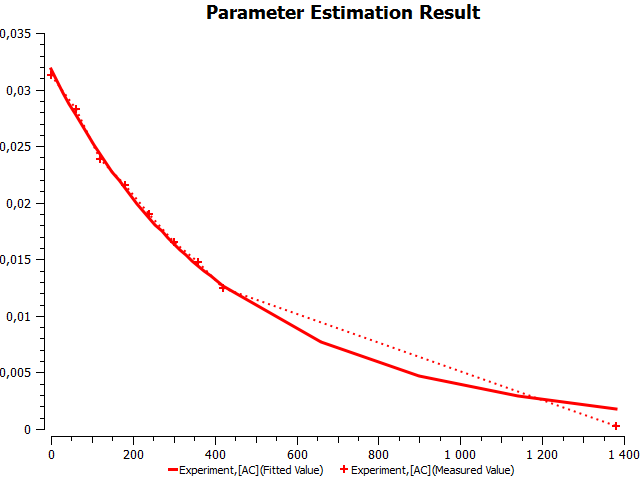

Supplement: RA-009-C9RA06124J-s013 [file RA-009-C9RA06124J-s013.tiff]

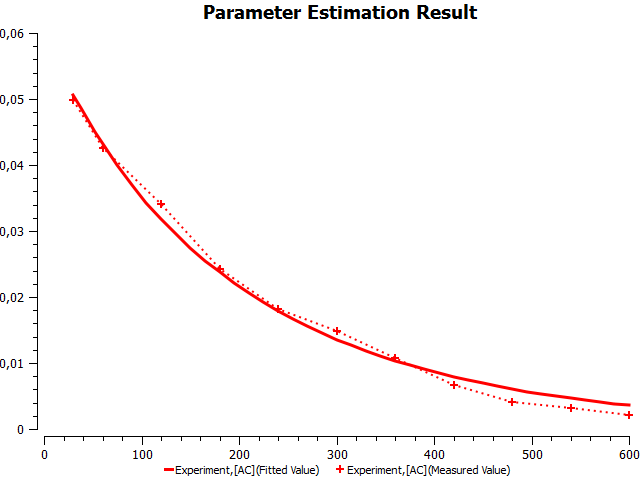

Supplement: RA-009-C9RA06124J-s016 [file RA-009-C9RA06124J-s016.tiff]

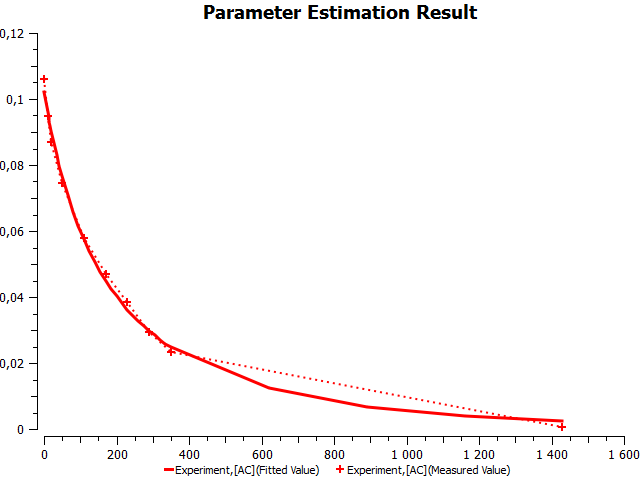

Supplement: RA-009-C9RA06124J-s019 [file RA-009-C9RA06124J-s019.tiff]

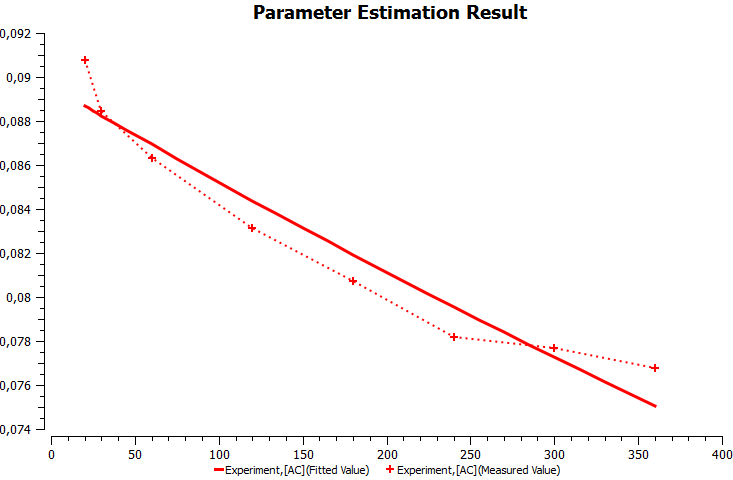

Supplement: RA-009-C9RA06124J-s022 [file RA-009-C9RA06124J-s022.tiff]

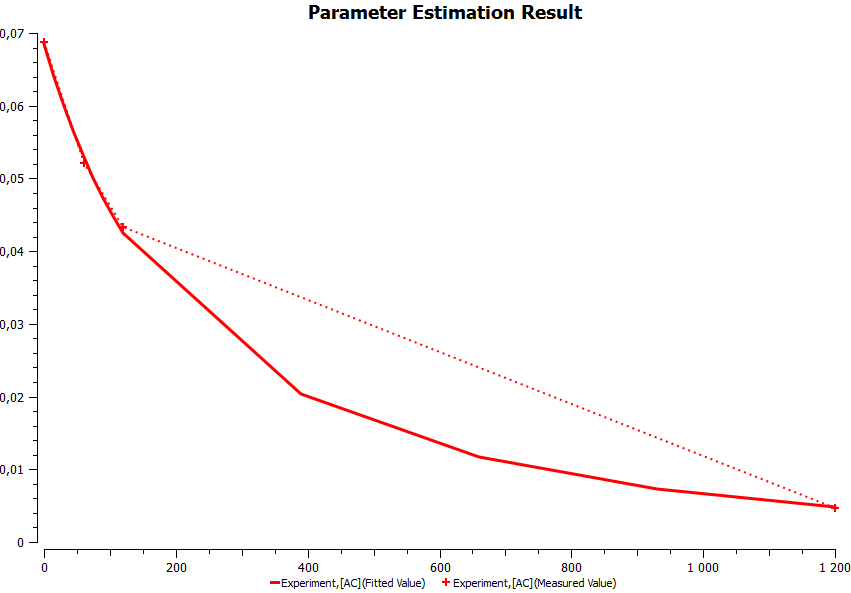

Supplement: RA-009-C9RA06124J-s025 [file RA-009-C9RA06124J-s025.tiff]

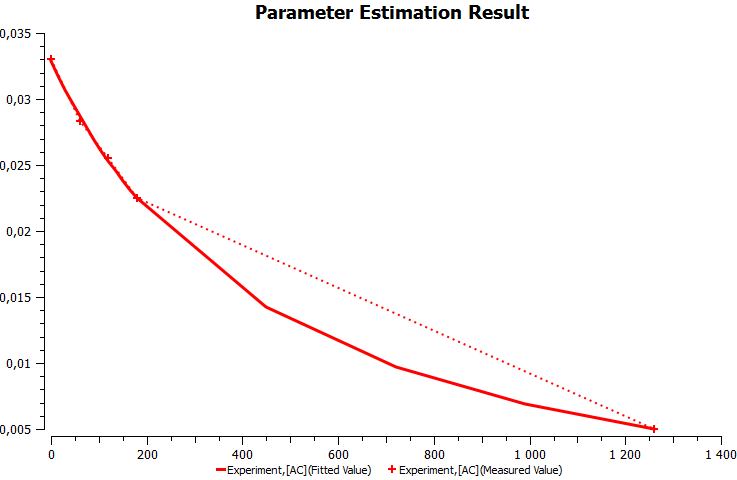

Supplement: RA-009-C9RA06124J-s028 [file RA-009-C9RA06124J-s028.tiff]

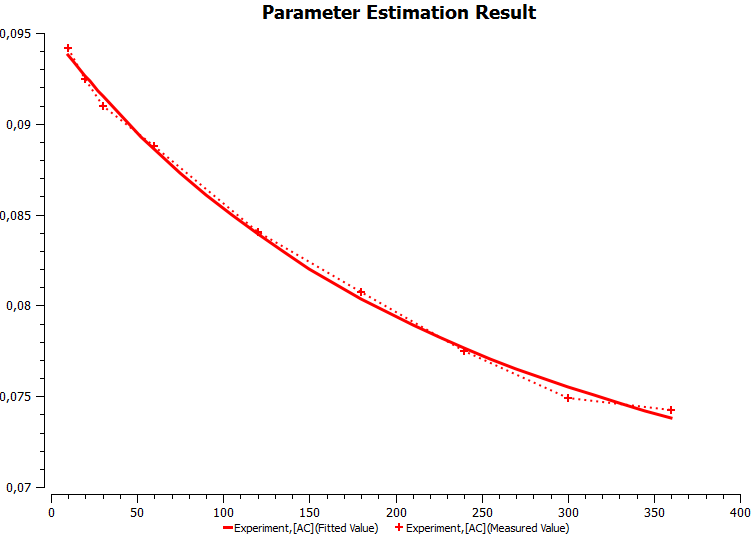

Supplement: RA-009-C9RA06124J-s031 [file RA-009-C9RA06124J-s031.tiff]
