# Supplementary material for: Unwanted hydrolysis or α/β-peptide bond formation: how long should the rate-limiting coupling step take?
Source: RSC Adv. 2019 Sep 27;9(53):30720–8. doi: 10.1039/c9ra06124j (PMC9072530; doi:10.1039/c9ra06124j)
Supplement: RA-009-C9RA06124J-s034 [file RA-009-C9RA06124J-s034.pdf]

Electronic Supplementary Material (ESI) for RSC Advances.
This journal is © The Royal Society of Chemistry 2019

Unwanted hydrolysis or α/β-peptide bond formation? How long the rate-limiting coupling step should take?

Viktória Goldschmidt Gőz,[1] Adrienn Nagy,[2] Viktor Farkas,[1] Ernő Keszei,[3] and András Perczel*[1,2]

[1]MTA-ELTE Protein Modeling Research Group, Pázmány P. sétány. 1/A, 1117 Budapest, Hungary

[2]Laboratory of Structural Chemistry and Biology, Institute of Chemistry, Eötvös Loránd University, Pázmány P. stny. 1/A, 1117 Budapest, Hungary

[3]Chemical Kinetics Laboratory, Institute of Chemistry, Eötvös Loránd University, Pázmány P. stny. 1/A, 1117 Budapest, Hungary

* *e-mail:* perczel.andras@ttk.elte.hu

Table of contents

1. Active ester formation with HOBt/DIC 2

2. ^{1}H-NMR Spectra and diagrams of the active esters formation and hydrolysis with PyBOP/DIEA 3

2.1. Fast hydrolyzing active esters 3

2.2. Slow hydrolyzing active esters 8

2.3. None hydrolyzing active esters 14

3. ^{1}H-NMR Spectra and diagrams of the active esters formation and hydrolysis with HOBt/DIC 20

4. Kinetic analysis 24

1. Active ester formation with HOBt/DIC

Table 1. Reaction times (min) and conversions (%) for the active ester formation (f) and hydrolysis (h) for Fmoc protected α- and β-amino acids with HOBt/DIC. Conversion after 10, 60 minutes and 3 hours are shown which is the limit of the couplings.

**Active esters with HOBt/DIC**	**Formation (f)**		**Hydrolysis (h)**		**Conversion (%) of formation/hydrolysis**		
	**t_f (min)**	**conversion (%)**	**t_h(min)**	**conversion (%)**	**10 min**	**60 min**	**180 min**
Fmoc-Val-OH	240	>99	∞	0	f: 22	f: 62	f: 95
Fmoc-Arg-OH	120	35	1440	100	f: 3	f: 17	f: 30
Fmoc-RibAFU(ip)-OH	60	55	1440	100	f: 28	f: 55	h: 46
Fmoc-GlcAPU(Me)-OH	120	73	∞	0	f: 21	f: 48	f: 73

2. ^{1}H-NMR Spectra and diagrams of the active esters formation and hydrolysis with PyBOP/DIEA

2.1. Fast hydrolyzing active esters

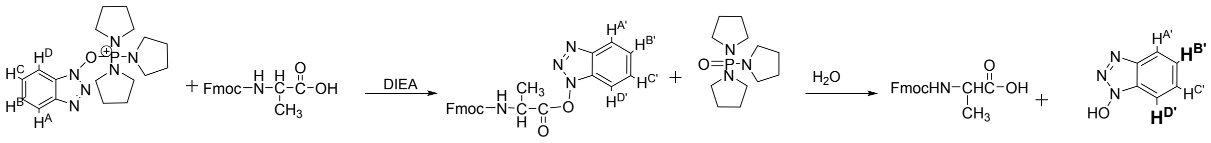

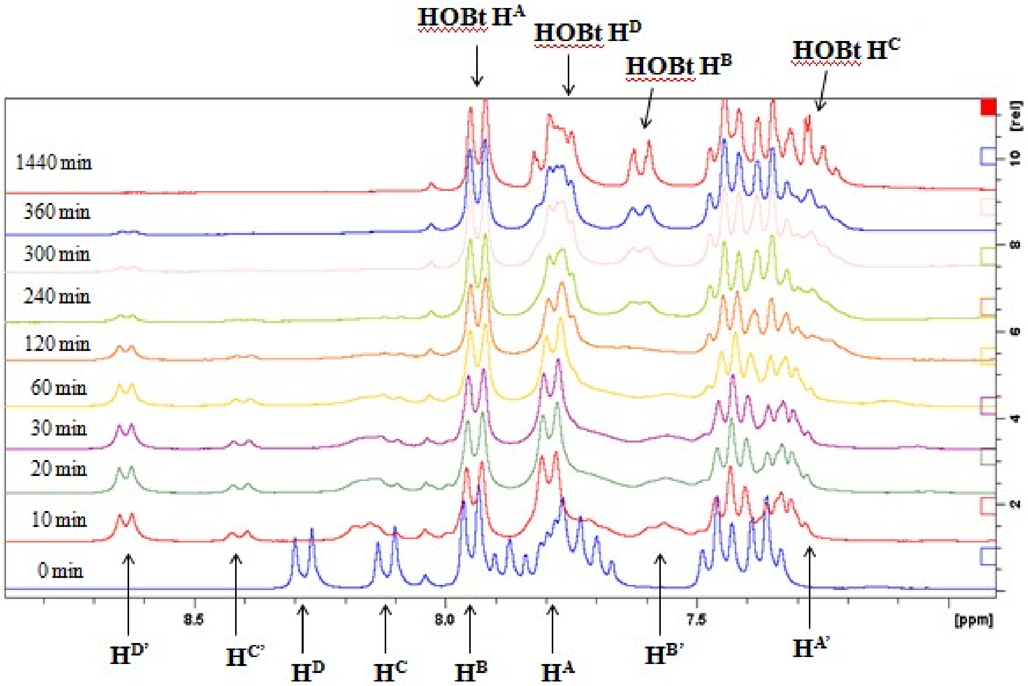

Figure 1. Reaction of Fmoc-Ala-OH and PyBOP/DIEA in DMF-d_7 as a function of time; ^{1}H NMR spectra, aromatic range, 250 MHz

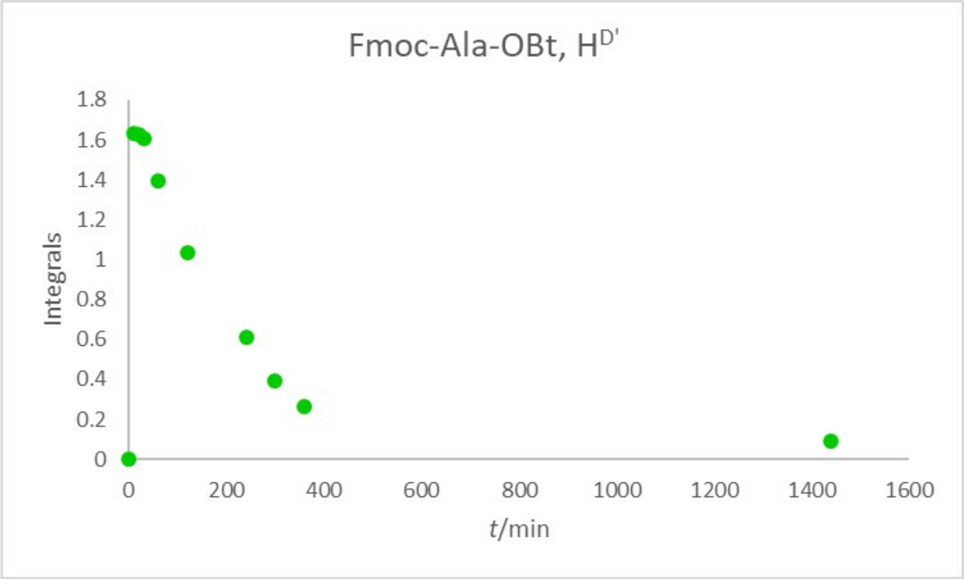

Figure 2. Integral-time diagram of Fmoc-Ala-OBt active ester, H$^{D'}$ signal at 8.63 ppm

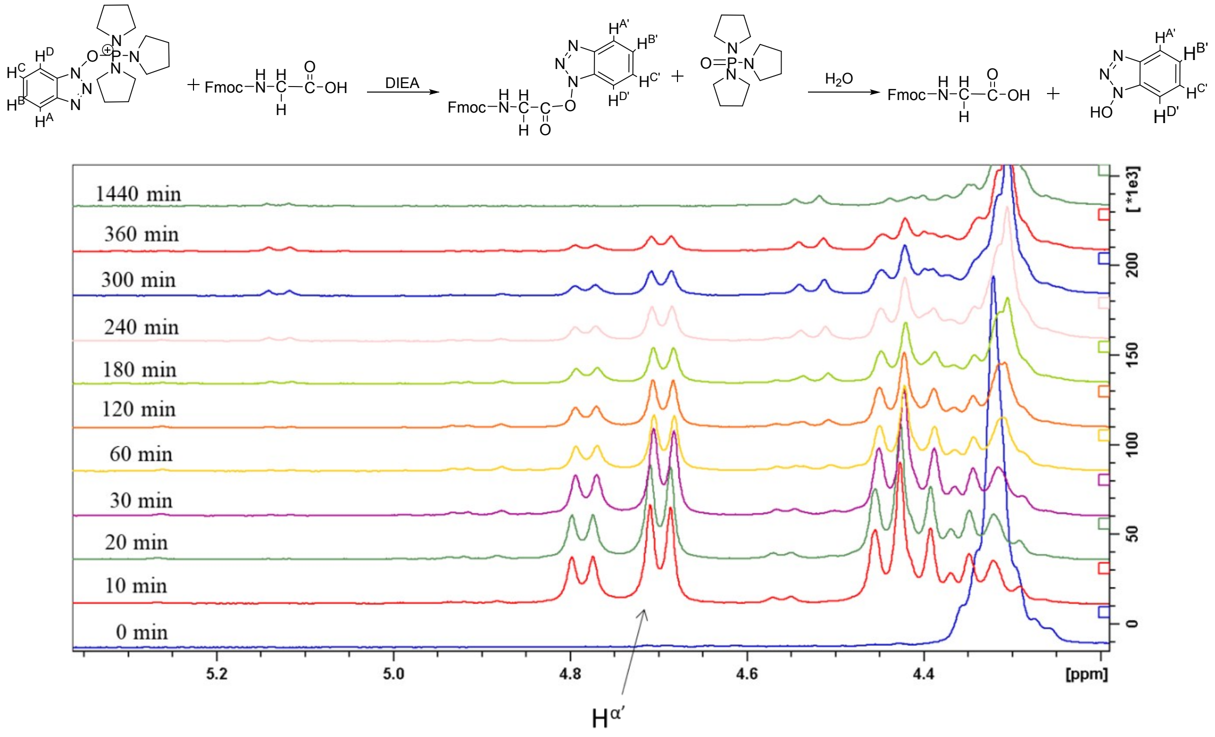

Figure 3. Reaction of Fmoc-Gly-OH and PyBOP/DIEA in DMF-d_7 as a function of time; ^{1}H NMR spectra, aliphatic region, 250 MHz

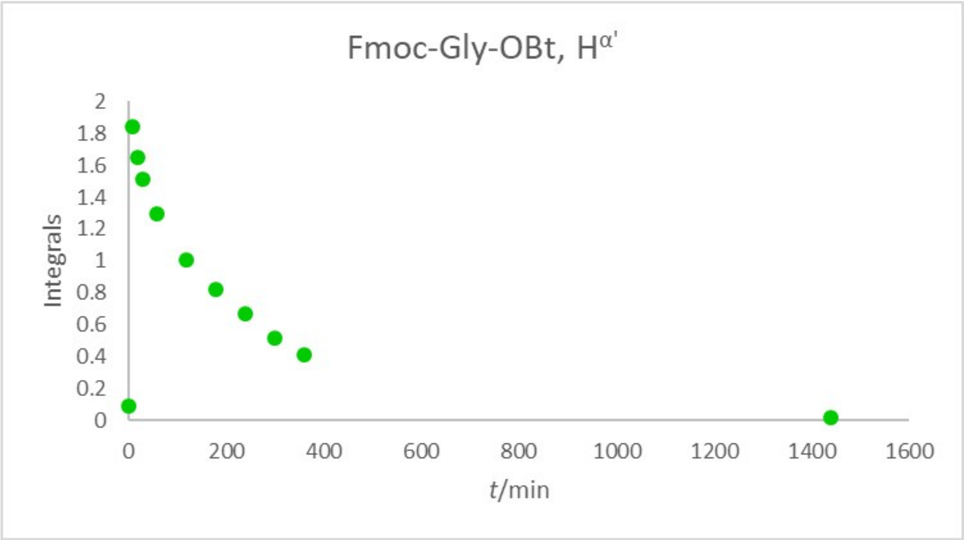

Figure 4. Integral-time diagram of Fmoc-Gly-OBt active ester, H^{α}' signal at 4.70 ppm

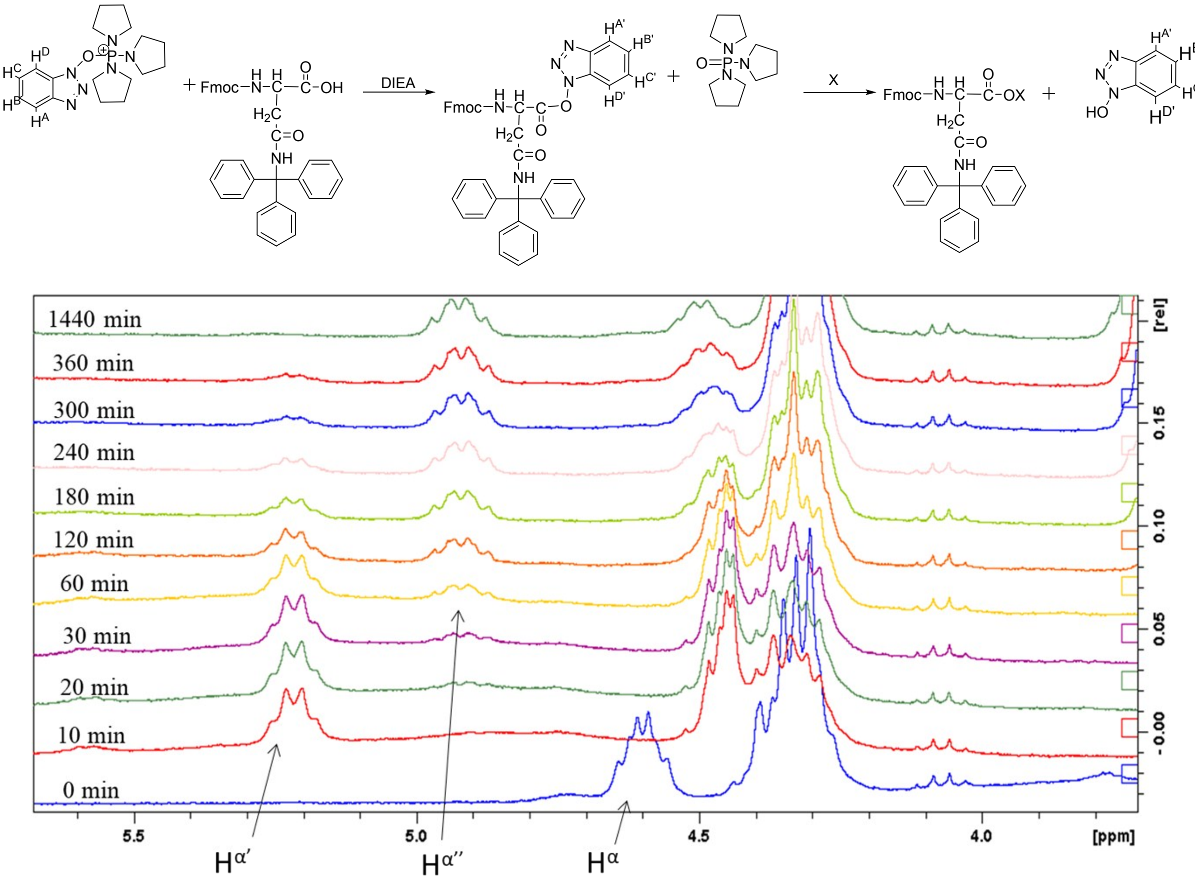

Figure 5. Reaction of Fmoc-Asn(Trt)-OH and PyBOP/DIEA in DMF-d_7 as a function of time; ^{1}H NMR spectra, aliphatic region, 250 MHz

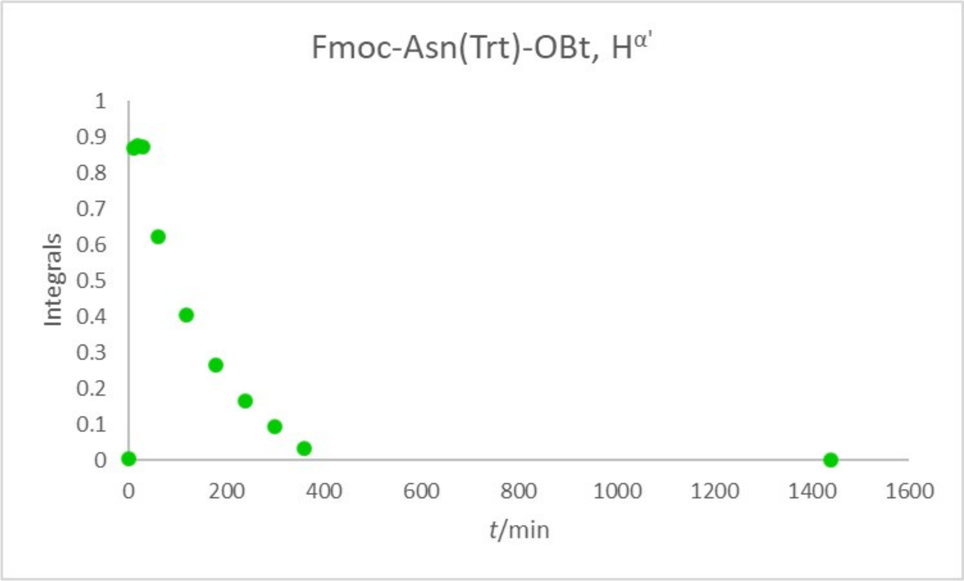

Figure 6. Integral-time diagram of Fmoc-Asn(Trt)-OBt active ester, $H^{\alpha'}$ signal at 5.21 ppm

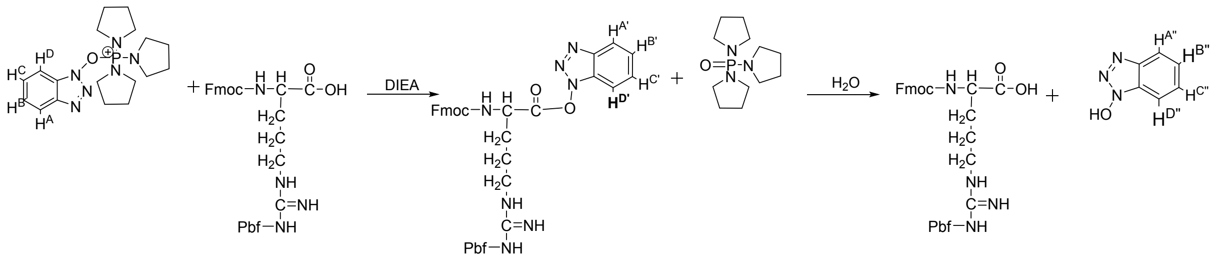

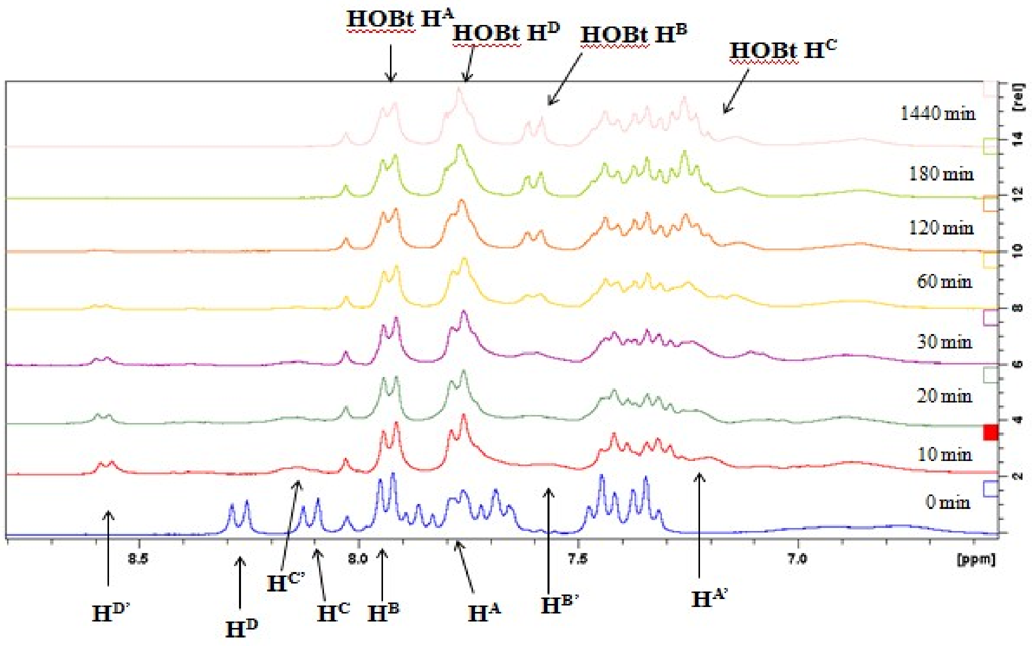

Figure 7. Reaction of Fmoc-Arg(Pbf)-OH and PyBOP/DIEA in DMF-d_7 as a function of time; ^{1}H NMR spectra, aromatic region, 250 MHz

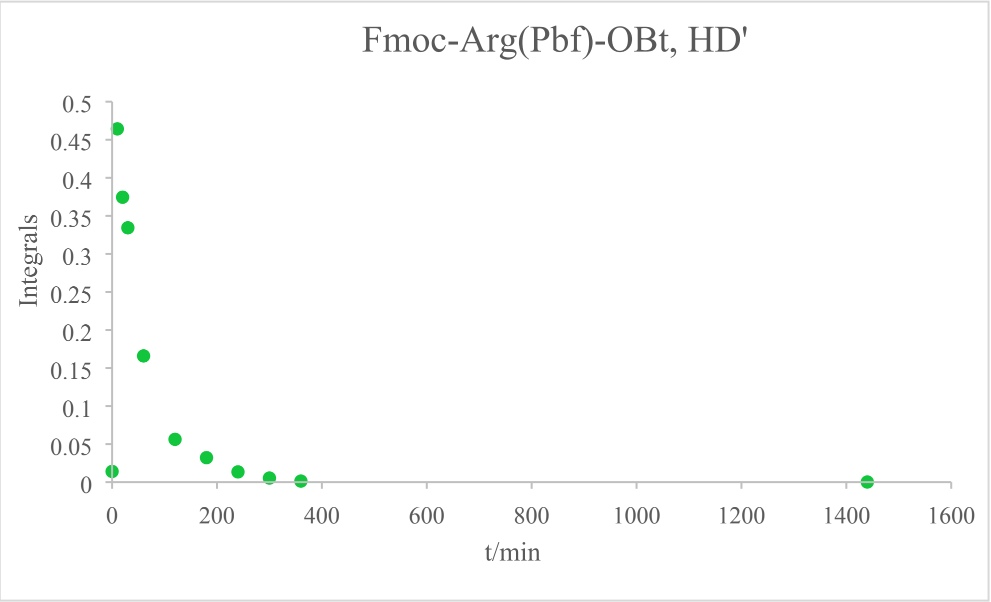

Figure 8. Integral-time diagram of Fmoc-Arg(Pbf)-OBt active ester, $H^{D'}$ signal at 8.62 ppm

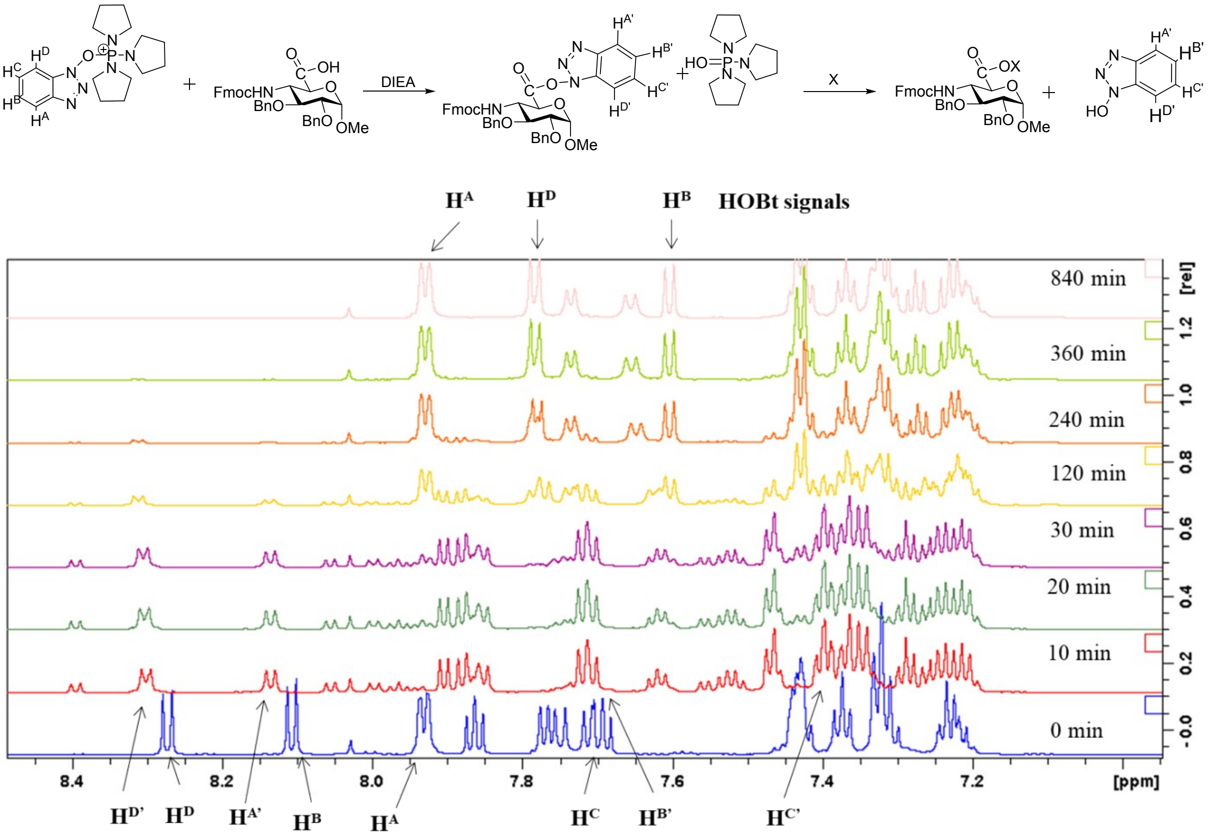

Figure 9. Reaction of Fmoc-GlcAPU(Me)-OH and PyBOP/DIEA in DMF-d_7 as a function of time; ^{1}H NMR spectra, aromatic region, 700 MHz

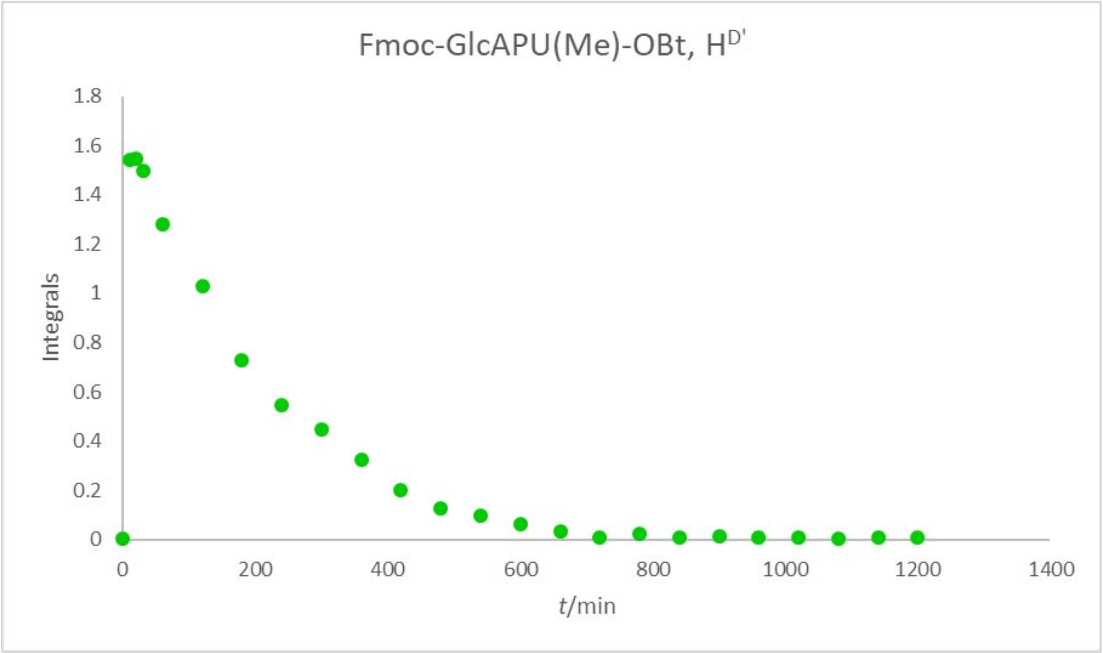

Figure 10. Integral-time diagram of Fmoc-GlcAPU(Me)-OBt active ester, $H^{D'}$ signal at 8.30 ppm

2.2. Slow hydrolyzing active esters

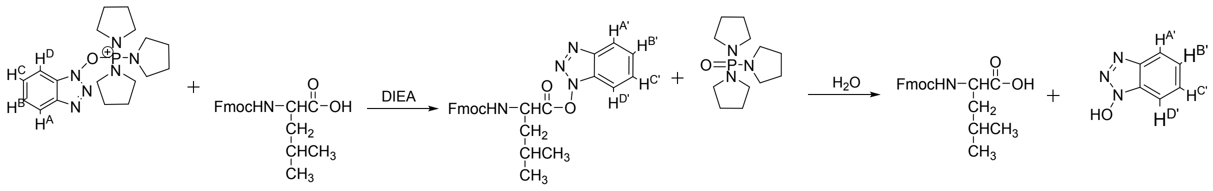

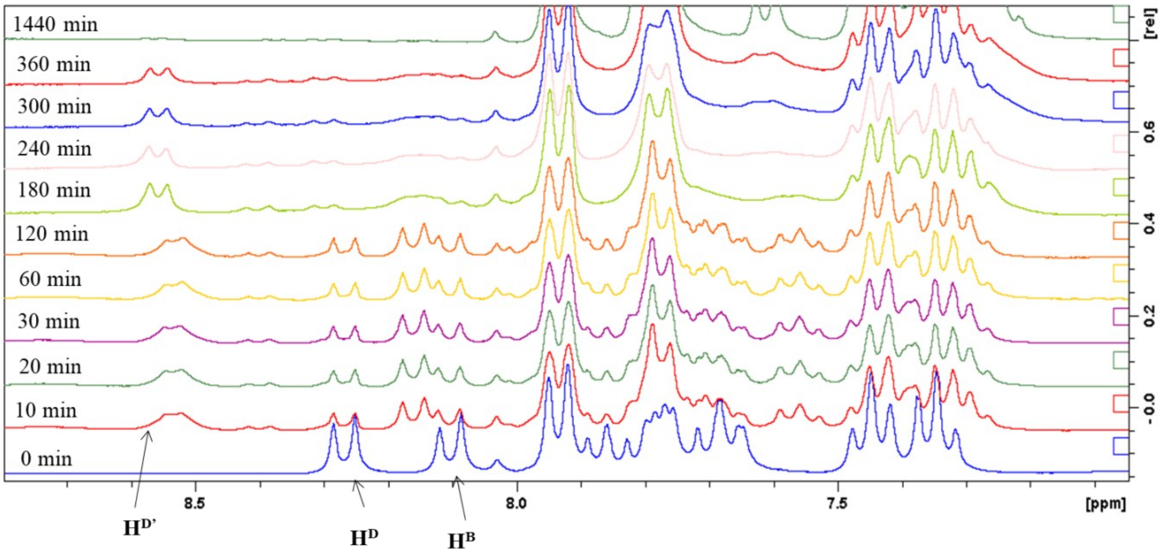

Figure 11. Reaction of Fmoc-Leu-OH and PyBOP/DIEA in DMF-d_7 as a function of time; ^{1}H NMR spectra, aromatic region, 250 MHz

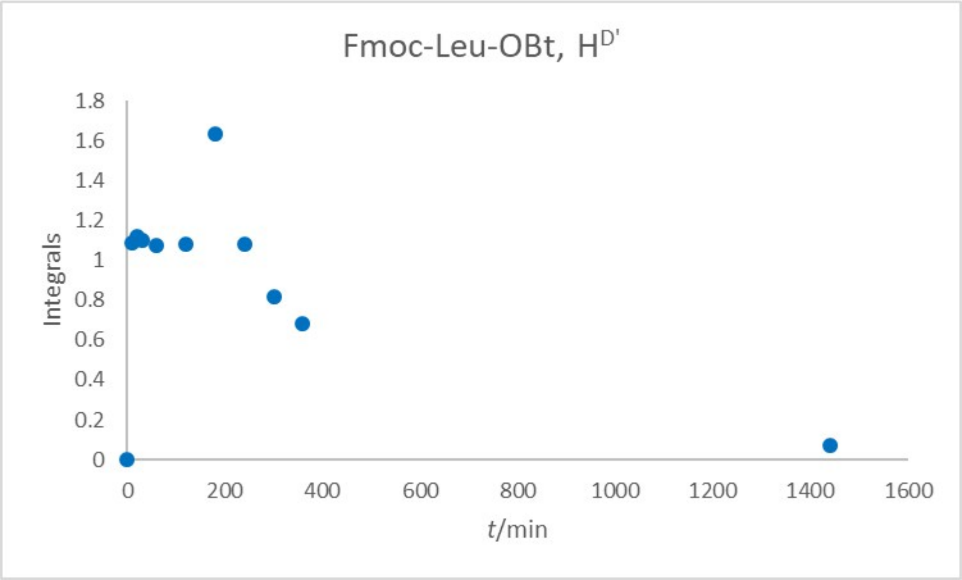

Figure 12. Integral-time diagram of Fmoc-Leu-OBt active ester, $H^{D'}$ signal at 8.53 ppm

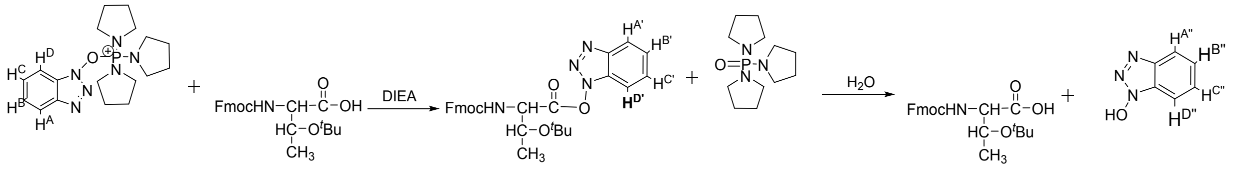

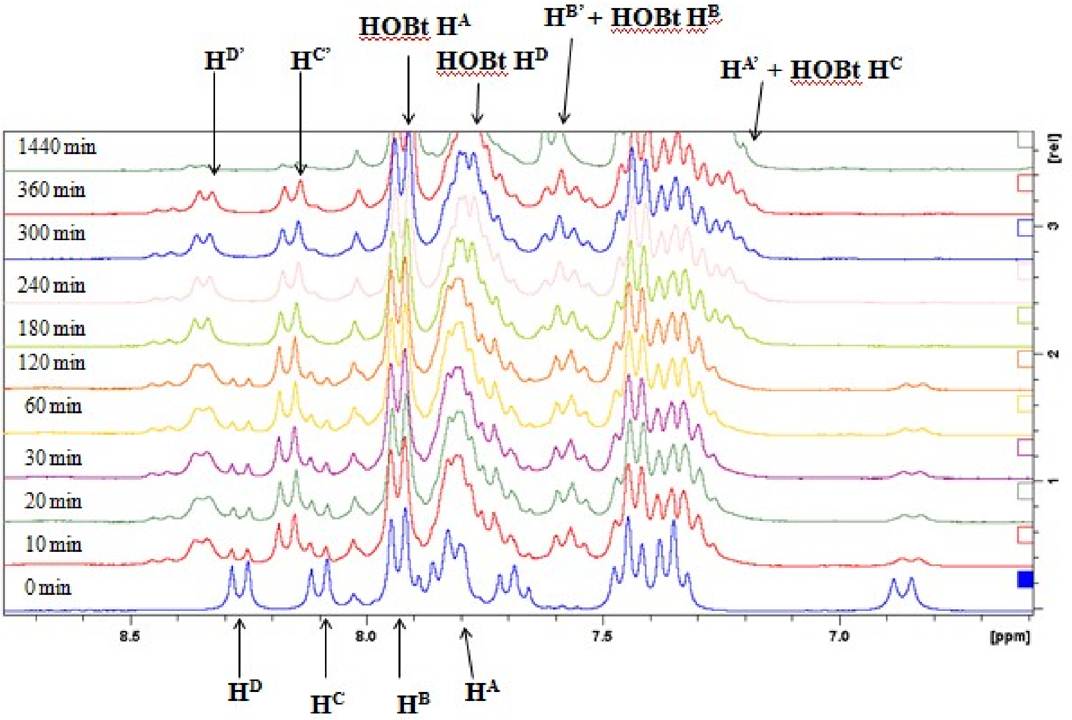

Figure 13. Reaction of Fmoc-Thr(^{t}Bu)-OH and PyBOP/DIEA in DMF-d_7 as a function of time; ^{1}H NMR spectra, aromatic region, 250 MHz

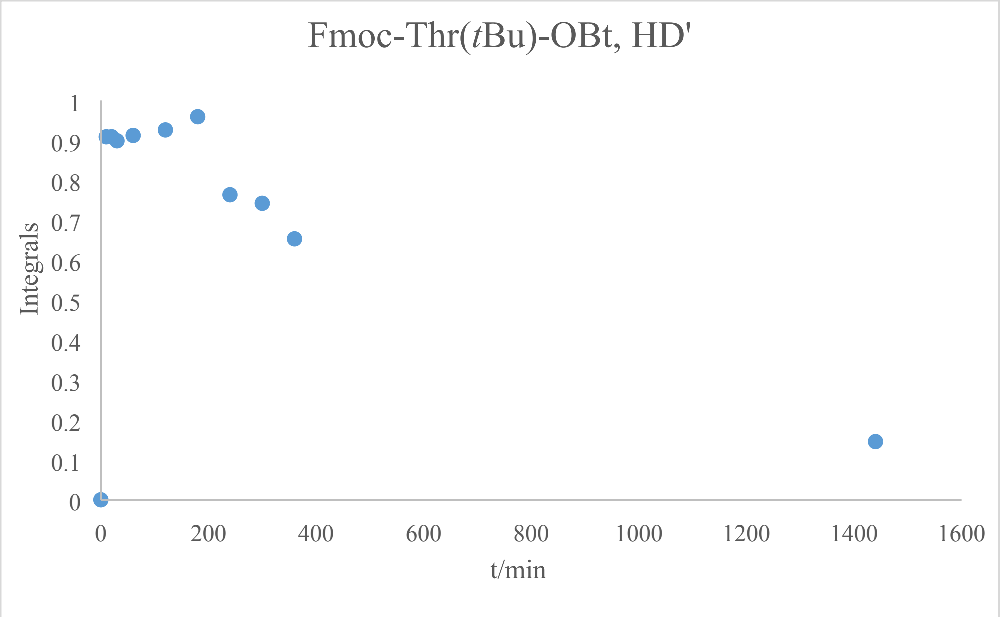

Figure 14. Integral-time diagram of Fmoc-Leu-OBt active ester, $H^{D'}$ signal at 8.35 ppm

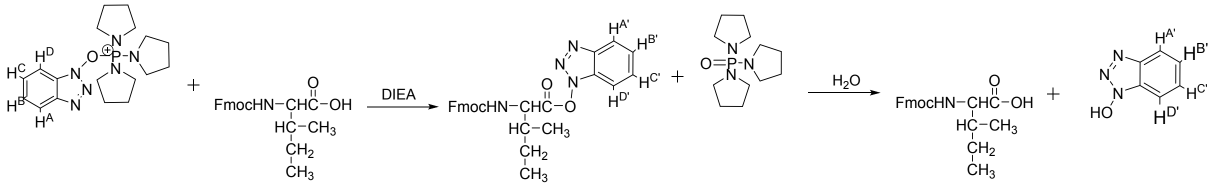

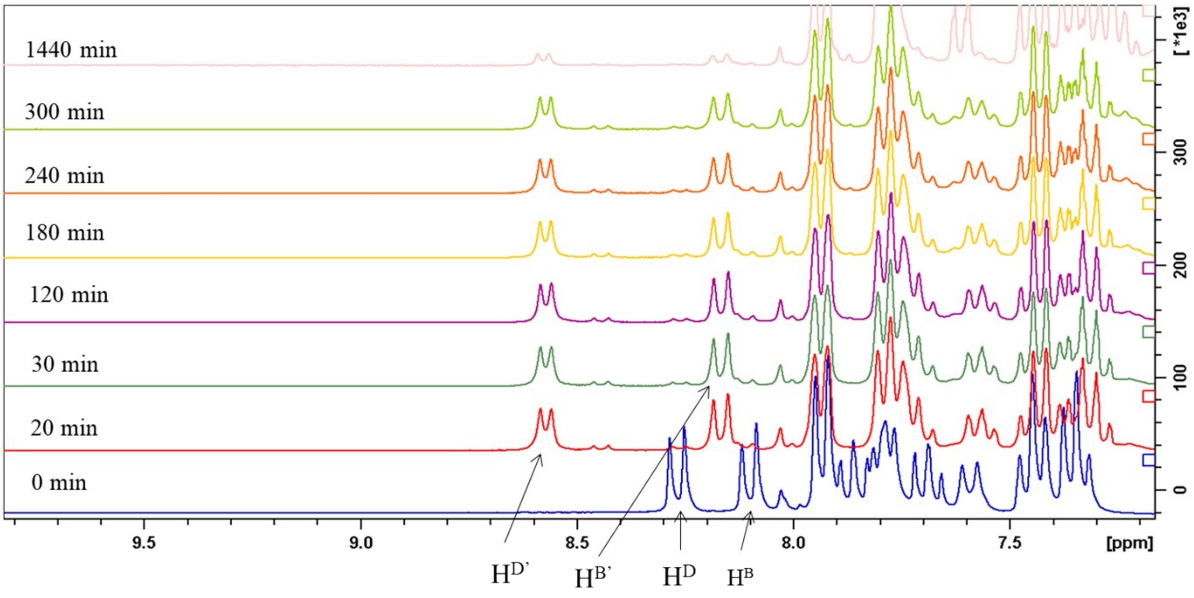

Figure 15. Reaction of Fmoc-Ile-OH and PyBOP/DIEA in DMF-d_7 as a function of time; ^{1}H NMR spectra, aromatic region, 250 MHz

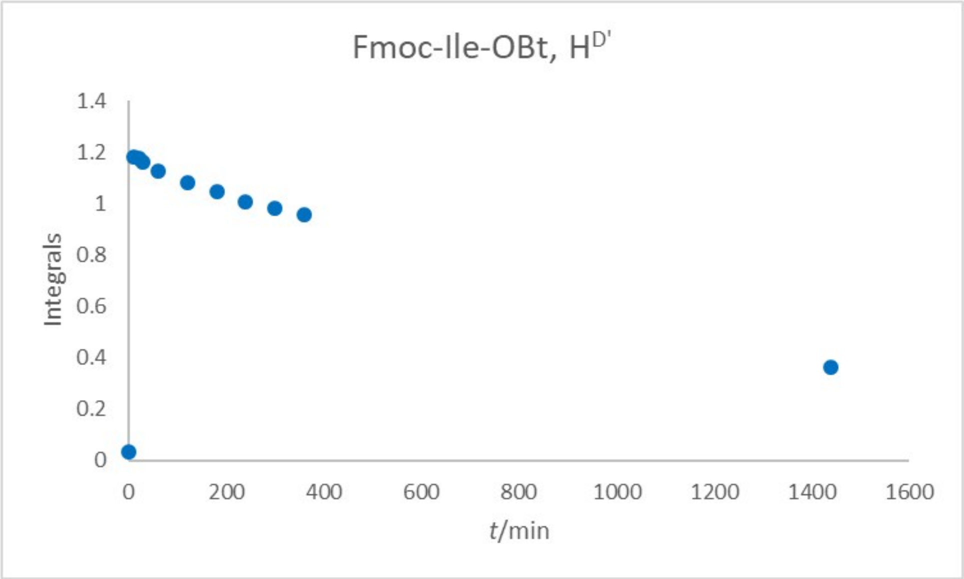

Figure 16. Integral-time diagram of Fmoc-Ile-OBt active ester, $H^{D'}$ signal at 8.57 ppm

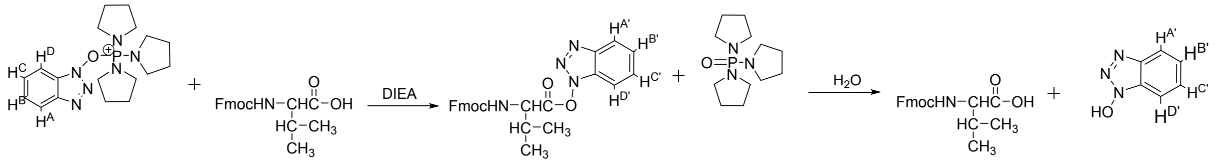

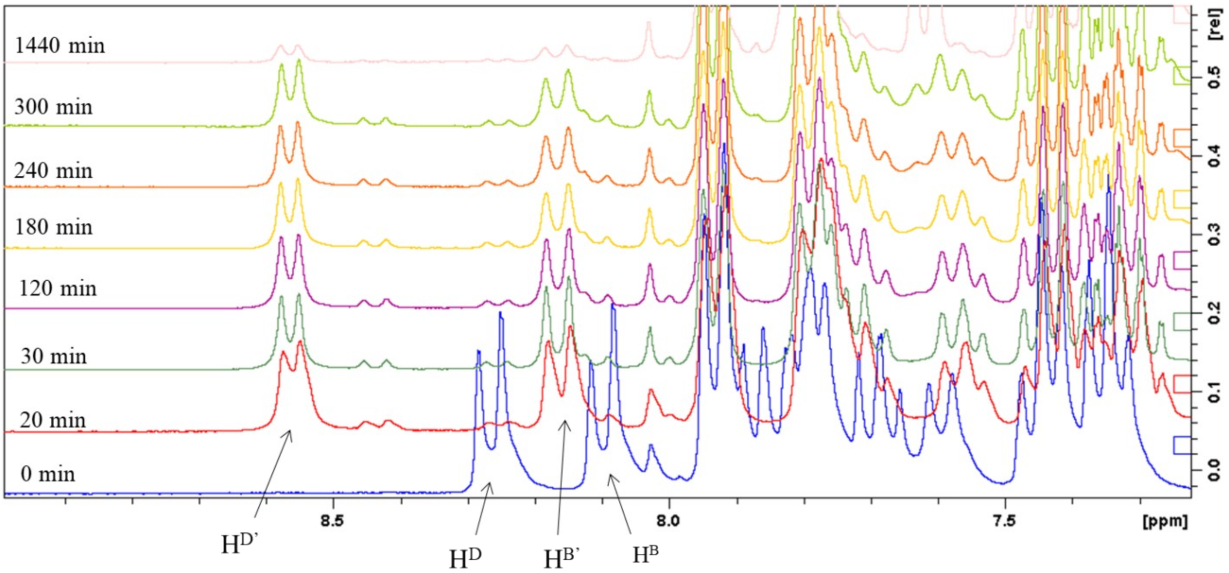

Figure 17. Reaction of Fmoc-Val-OH and PyBOP/DIEA in DMF-d_7 as a function of time; ^{1}H NMR spectra, aromatic region, 250 MHz

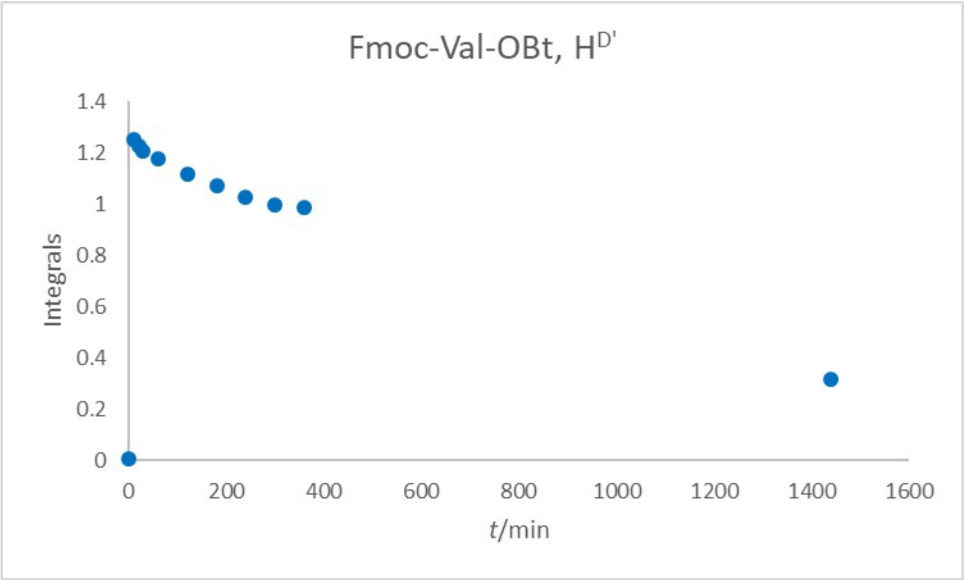

Figure 18. Integral-time diagram of Fmoc-Val-OBt active ester, H$^{D'}$ signal at 8.56 ppm

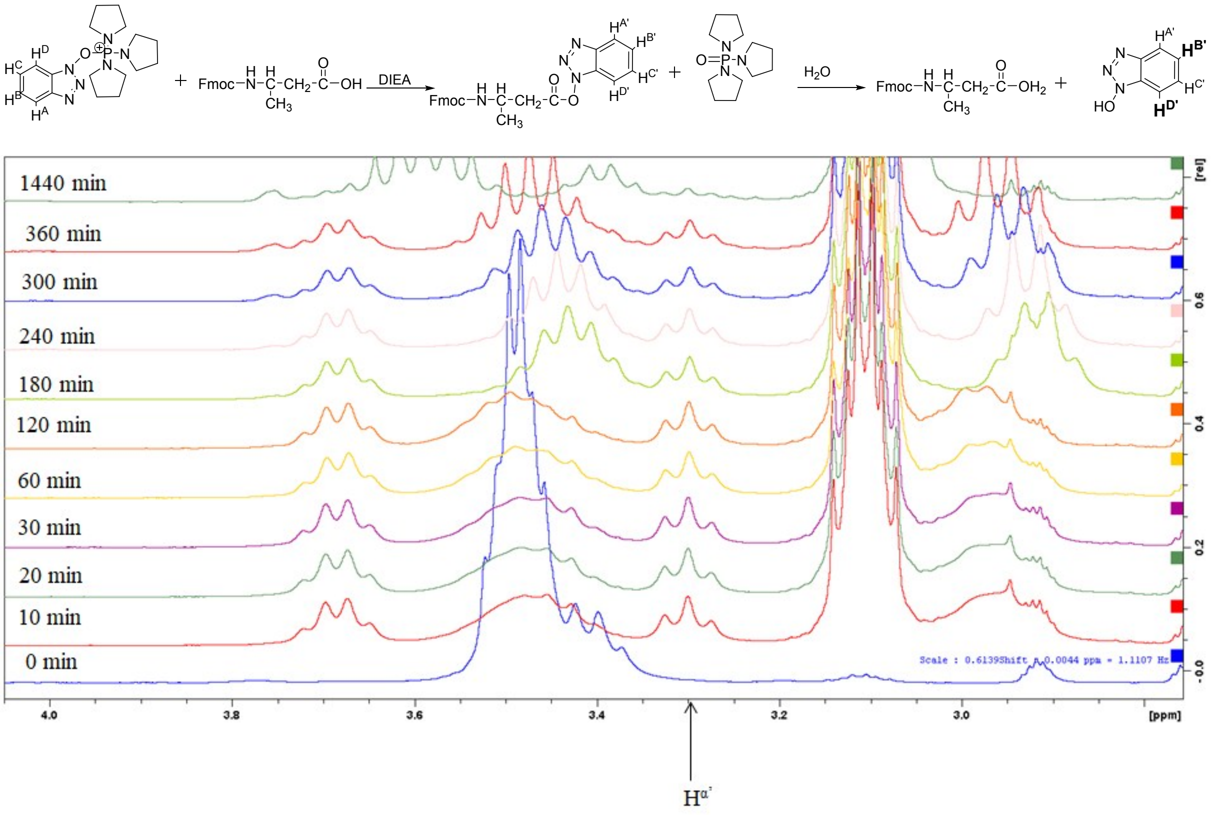

Figure 19. Reaction of Fmoc-β-Ala-OH and PyBOP/DIEA in DMF-d_7 as a function of time; ^1H NMR spectra, aliphatic region, 250 MHz

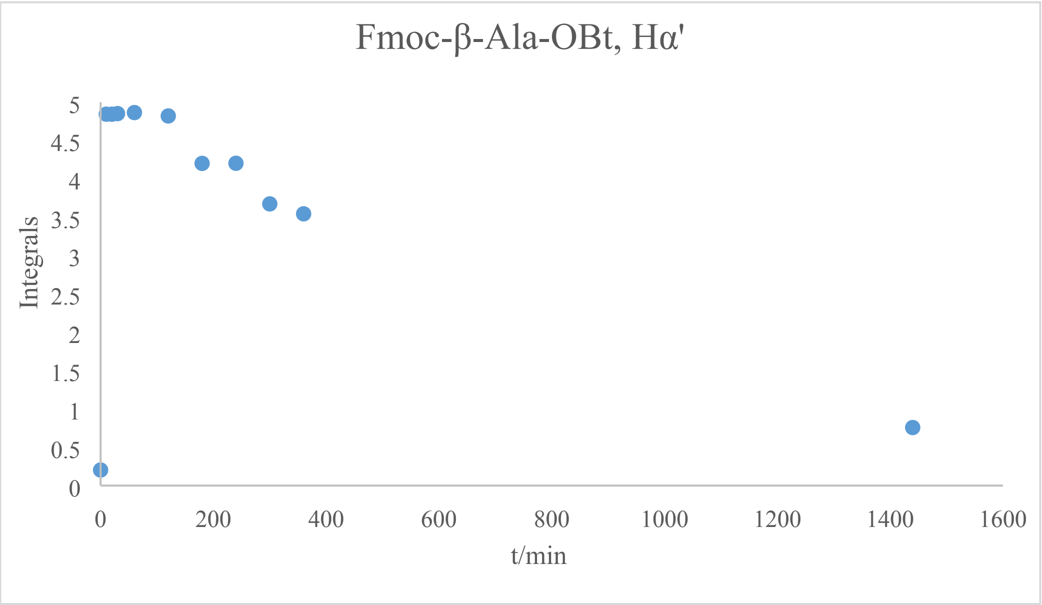

Figure 20. Integral-time diagram of Fmoc-β-Ala-OBt active ester, $H^{\alpha'}$ signal at 3.3 ppm

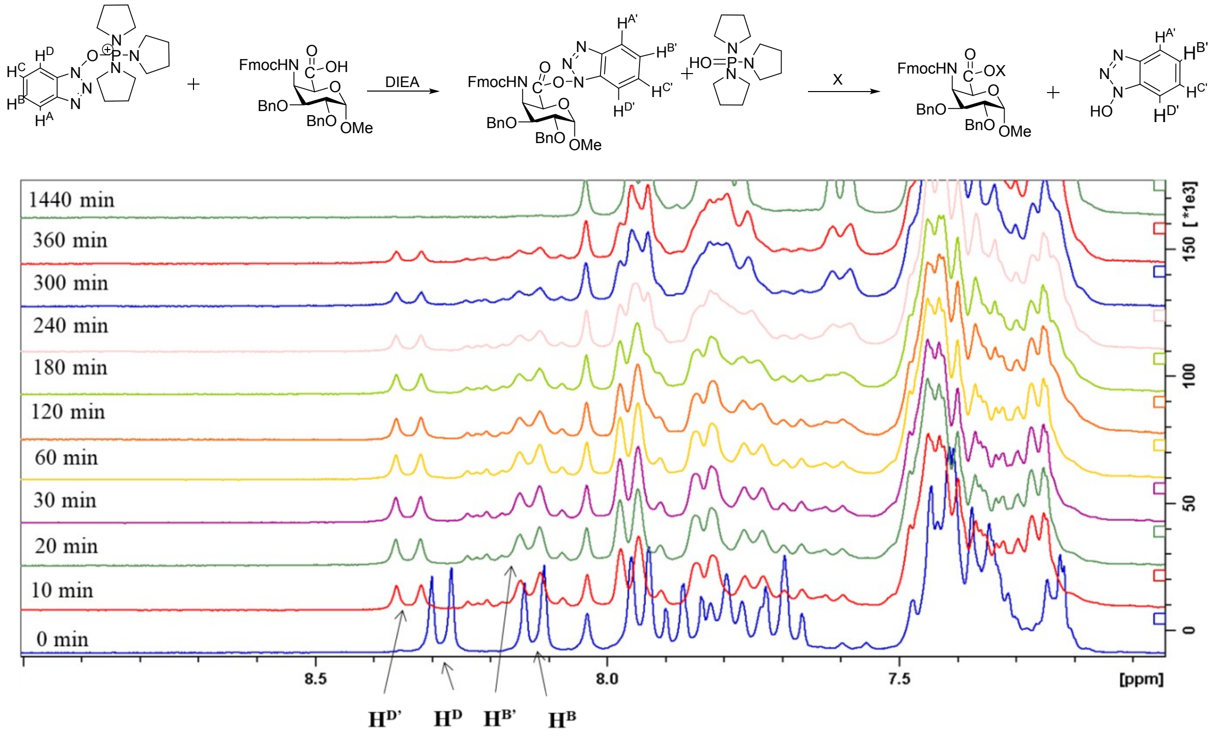

Figure 21. Reaction of Fmoc-GalAPU(Me)-OH and PyBOP/DIEA in DMF-d_7 as a function of time; ^{1}H NMR spectra, aromatic region, 250 MHz

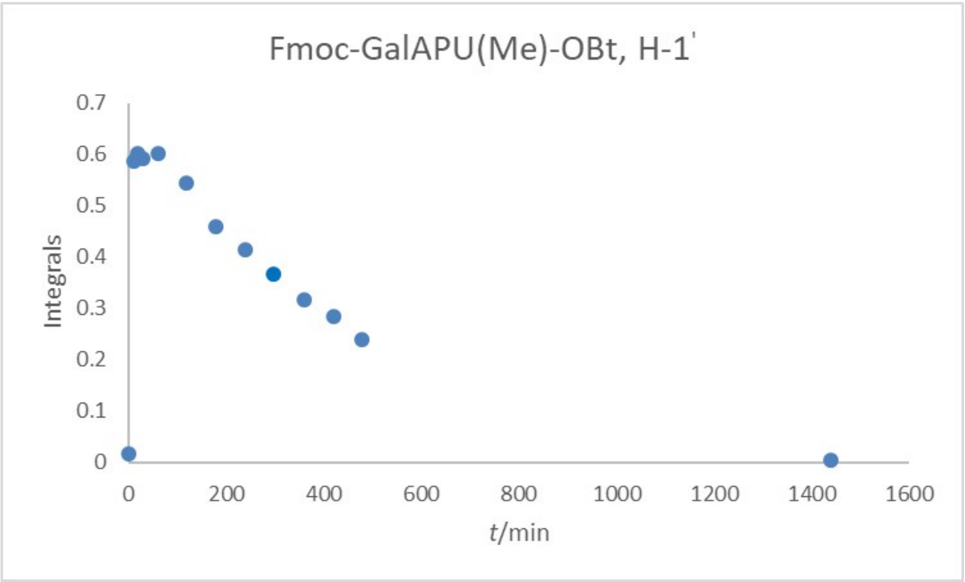

Figure 22. Integral-time diagram of Fmoc-GalAPU(Me)-OBt active ester, H-1’ signal at 5.53 ppm

2.3. None hydrolyzing active esters

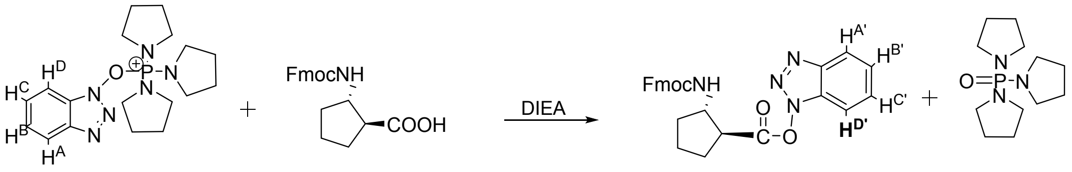

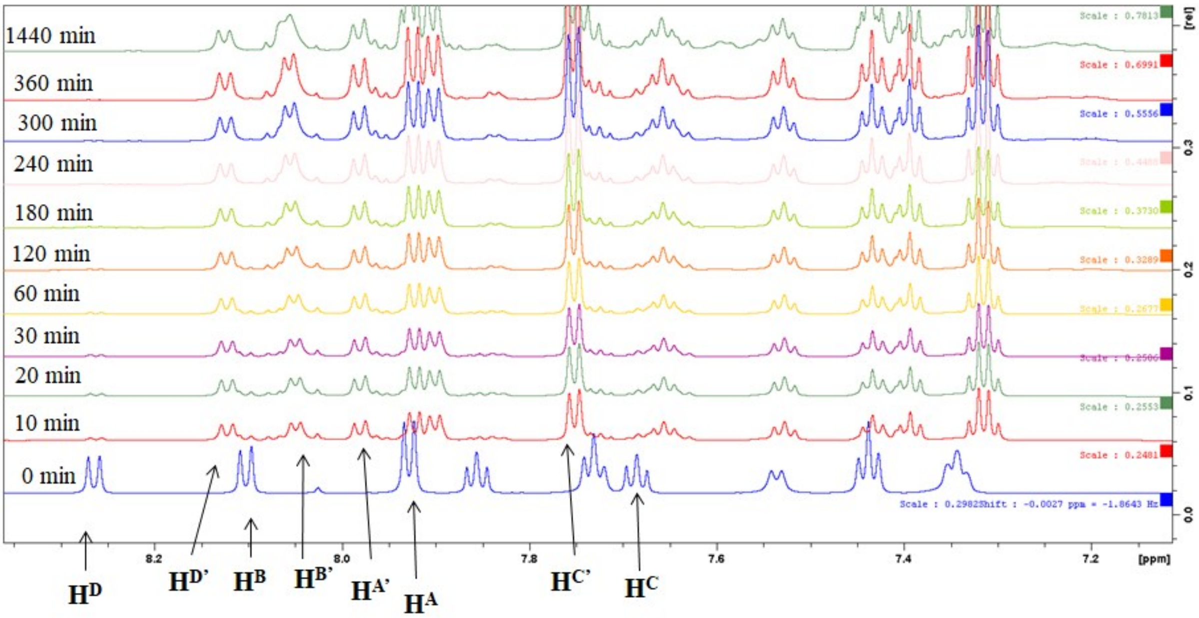

Figure 23. Reaction of Fmoc-ACPC-OH and PyBOP/DIEA in DMF-d_7 as a function of time; ^{1}H NMR spectra, aromatic region, 700 MHz

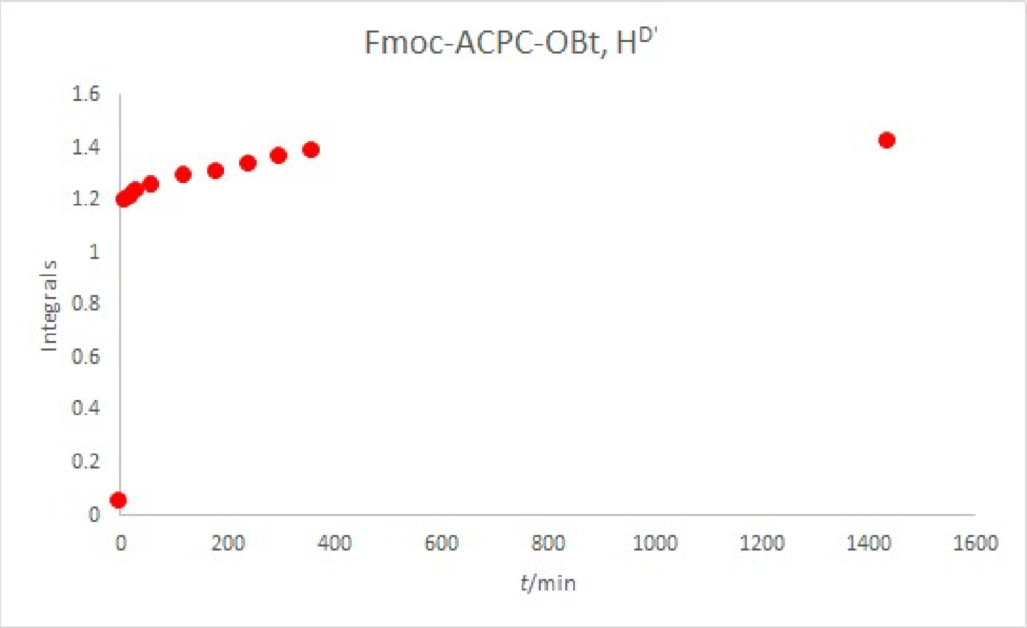

Figure 24. Integral-time diagram of Fmoc-ACPC-OBt active ester, $H^{D'}$ signal at 8.13 ppm

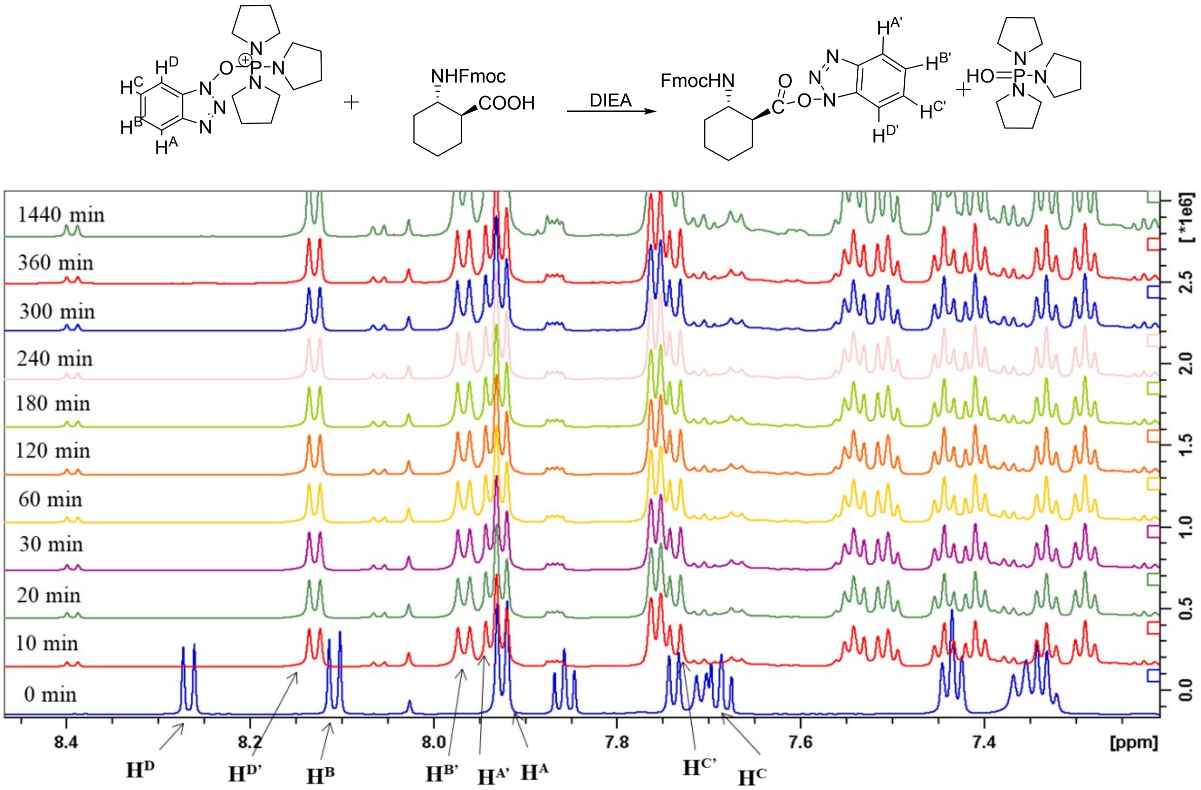

Figure 25. Reaction of Fmoc-ACHC-OH and PyBOP/DIEA in DMF-d_7 as a function of time; ^{1}H NMR spectra, aromatic region, 700 MHz

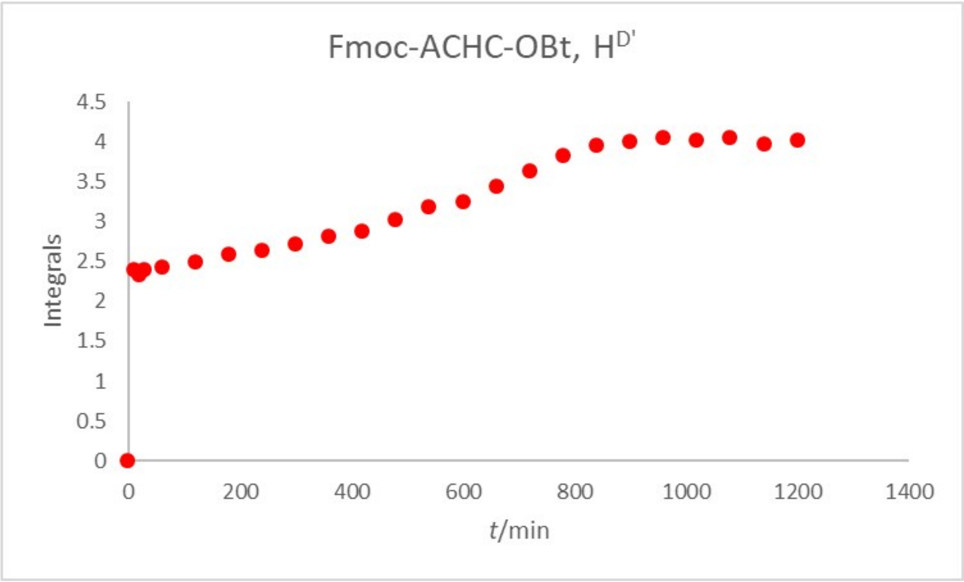

Figure 26. Integral-time diagram of Fmoc-ACPC-OBt active ester, H-1’ signal at 8.13 ppm

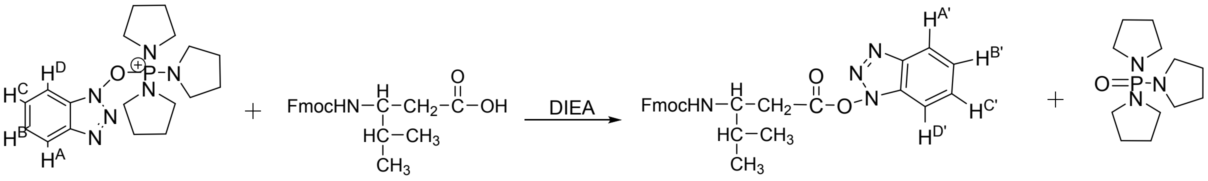

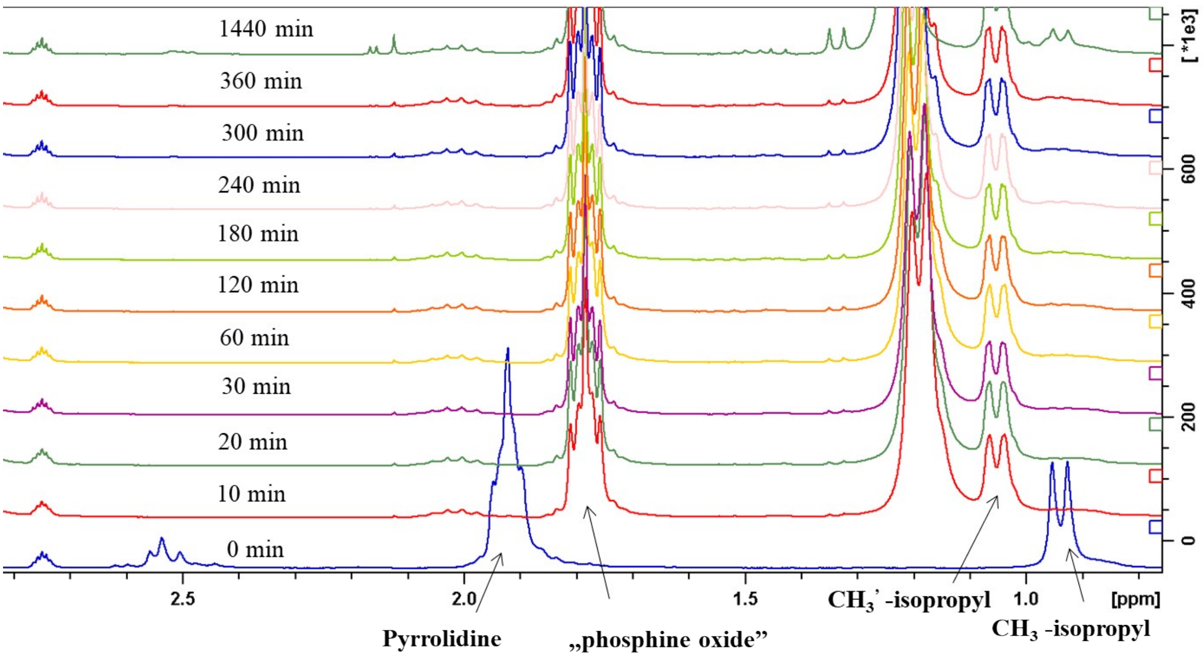

Figure 27. Reaction of Fmoc-β[3]-Val-OH and PyBOP/DIEA in DMF-d_7 as a function of time; ^1H NMR spectra, side-chain region, 250 MHz

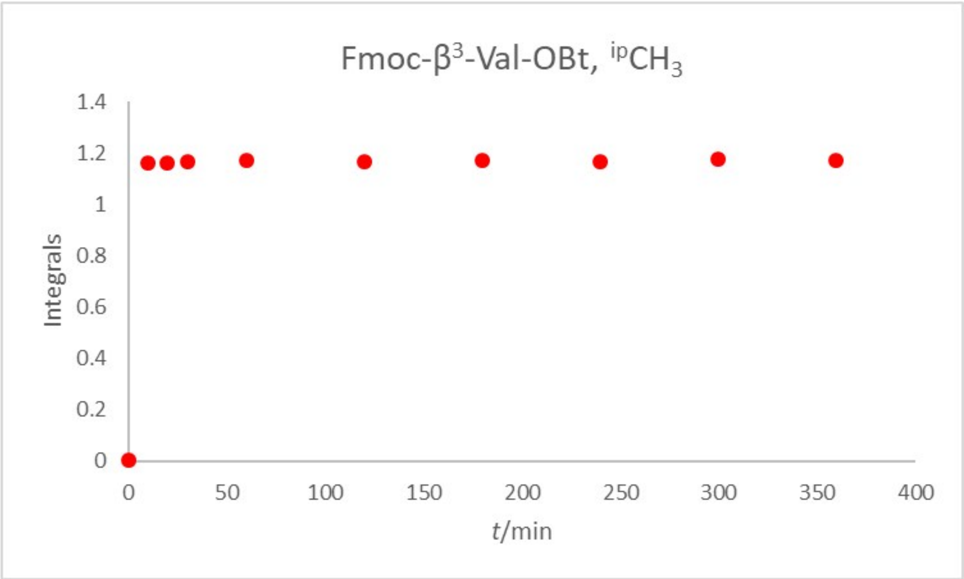

Figure 28. Integral-time diagram of Fmoc-β[3]-Val-OBt active ester, $^{ip}CH_3$ signal at 1.04 ppm

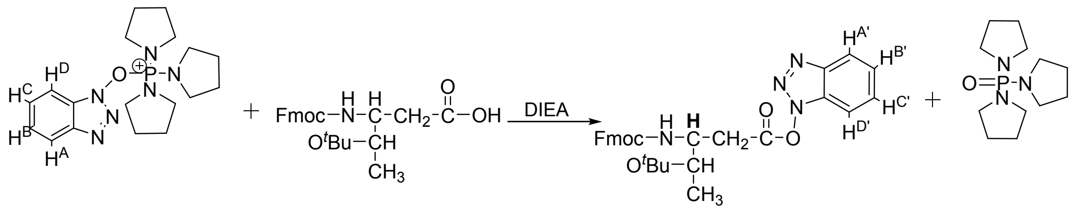

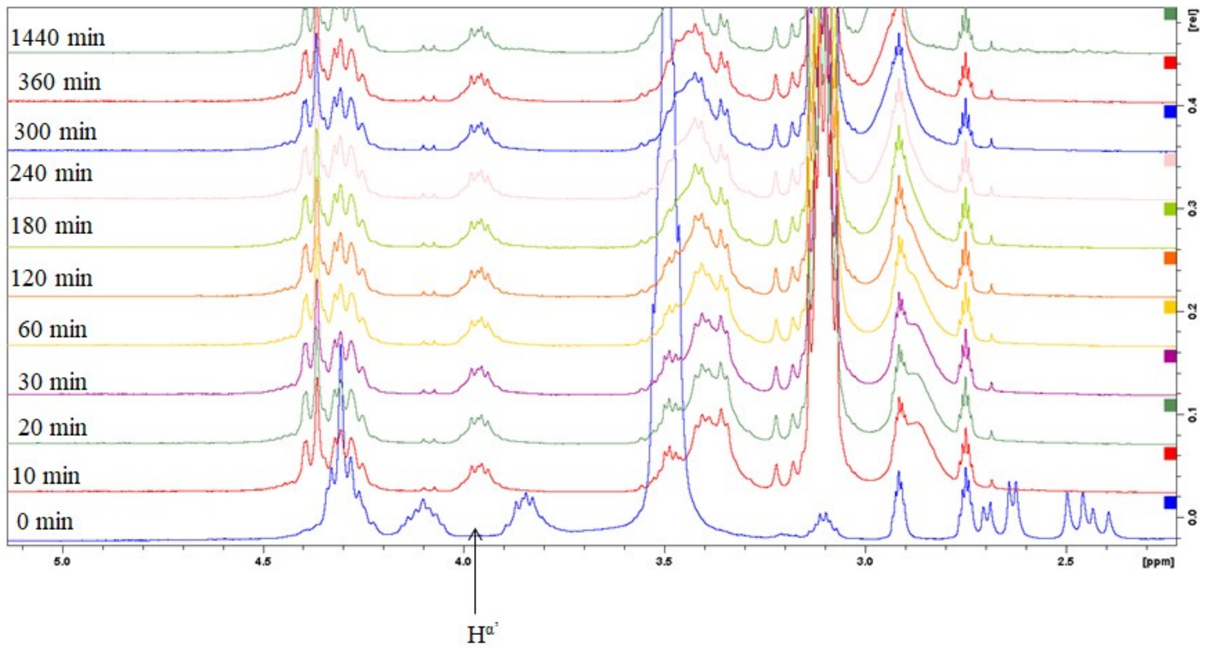

Figure 29. Reaction of Fmoc-β[3]-Thr(^{t}Bu)-OH and PyBOP/DIEA in DMF-d_7 as a function of time; ^{1}H NMR spectra, aliphatic region, 250 MHz

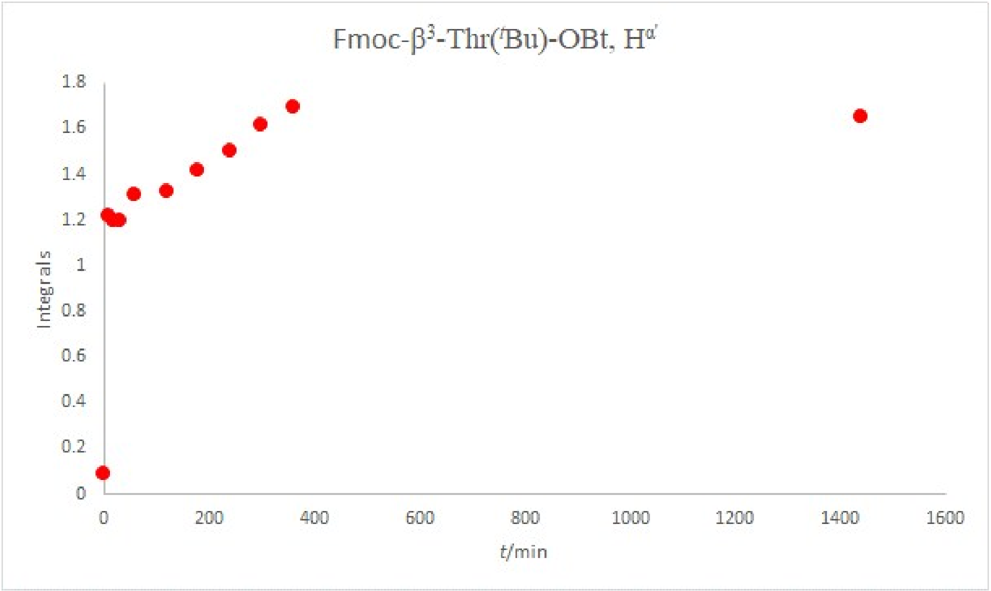

Figure 30. Integral-time diagram of Fmoc-β[3]-Thr(^{t}Bu)-OBt active ester, $H^{\alpha'}$ signal at 3.98 ppm

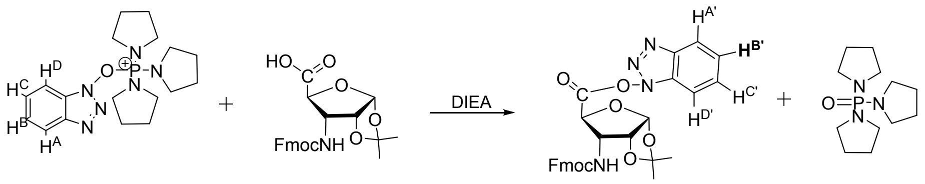

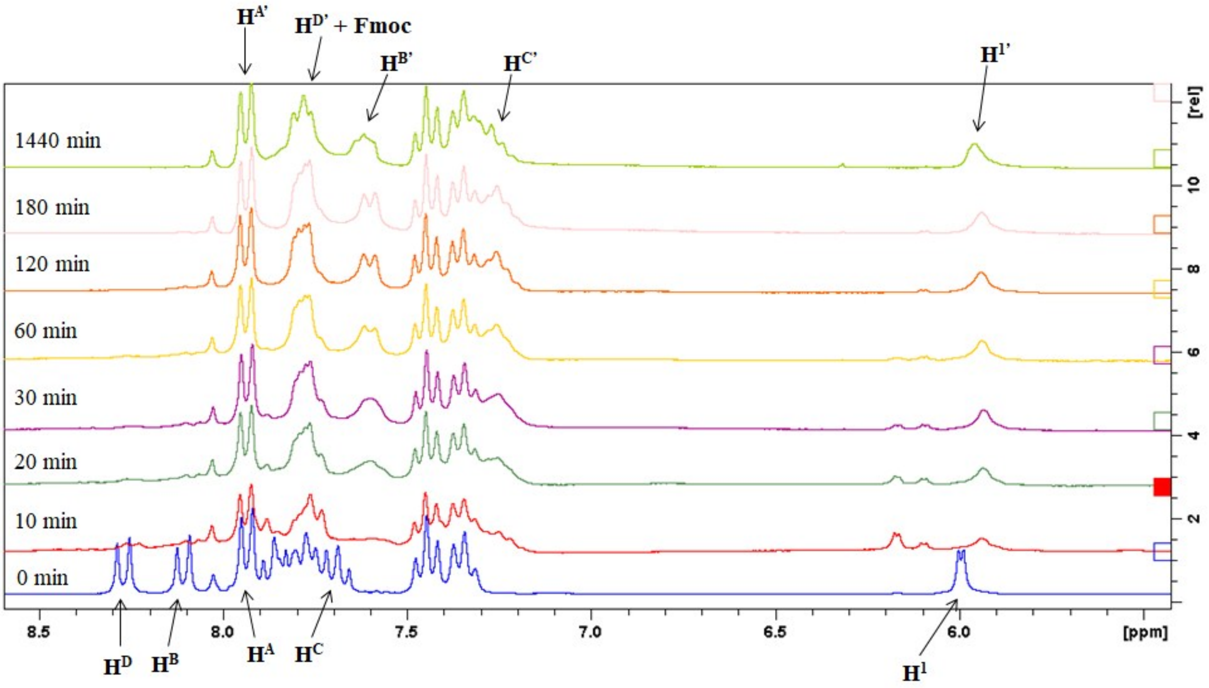

Figure 31. Reaction of Fmoc-RibAFU(ip)-OH and PyBOP/DIEA in DMF-d$_7$ as a function of time; ^{1}H NMR spectra, aromatic region, 250 MHz

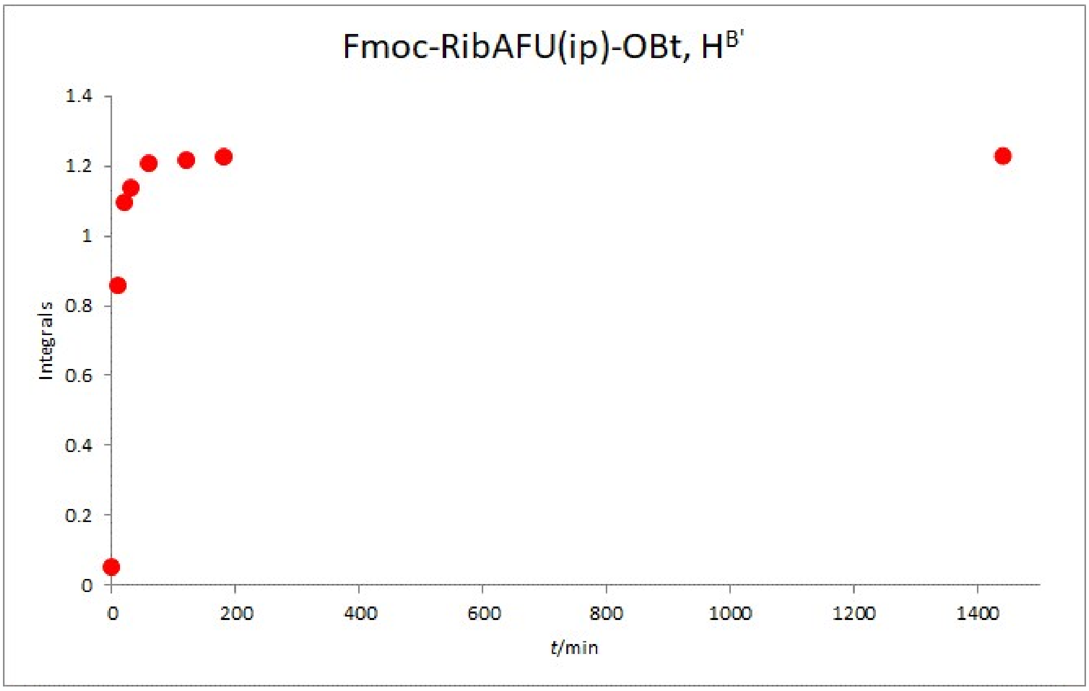

Figure 32. Integral-time diagram of Fmoc-RibAFU(ip)-OBt active ester, H$^{B'}$ signal at 7.61 ppm

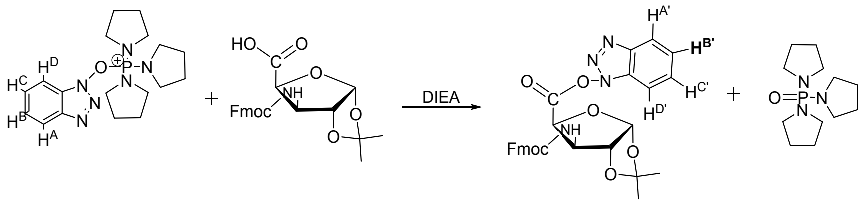

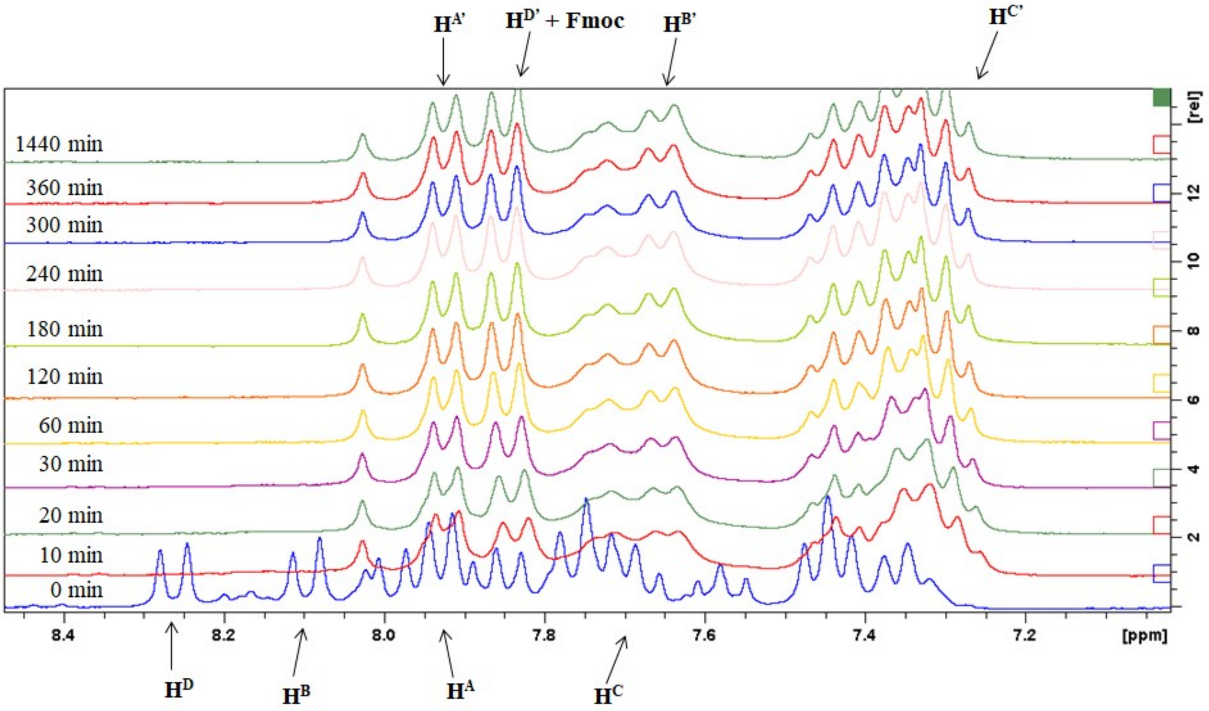

Figure 33. Reaction of Fmoc-XylAFU(ip)-OH and PyBOP/DIEA in DMF-d$_7$ as a function of time; ^{1}H NMR spectra, aromatic region, 250 MHz

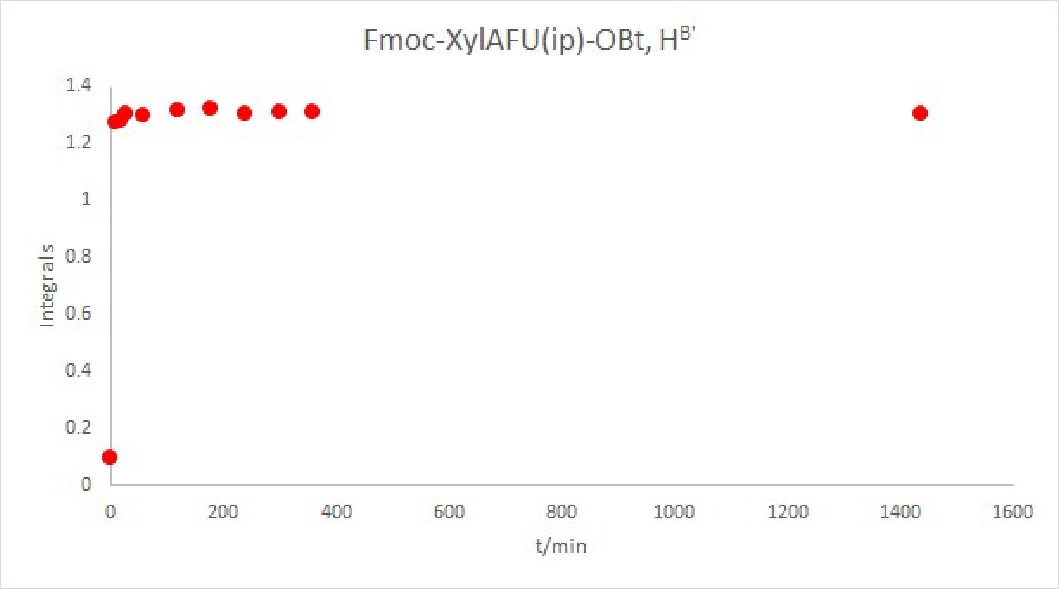

Figure 34. Integral-time diagram of Fmoc-XylAFU(ip)-OBt active ester, H$^{B'}$ signal at 7.65 ppm

3. ^{1}H-NMR Spectra and diagrams of the active esters formation and hydrolysis with HOBt/DIC

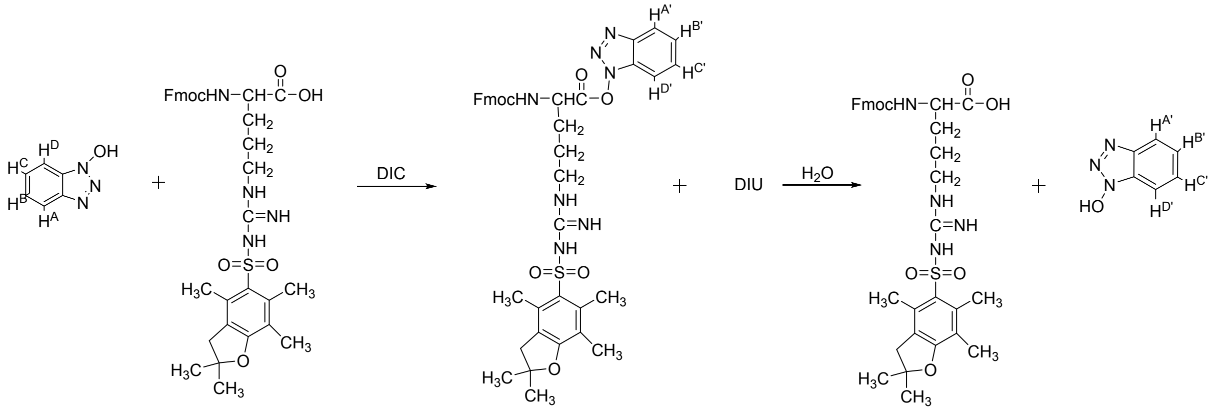

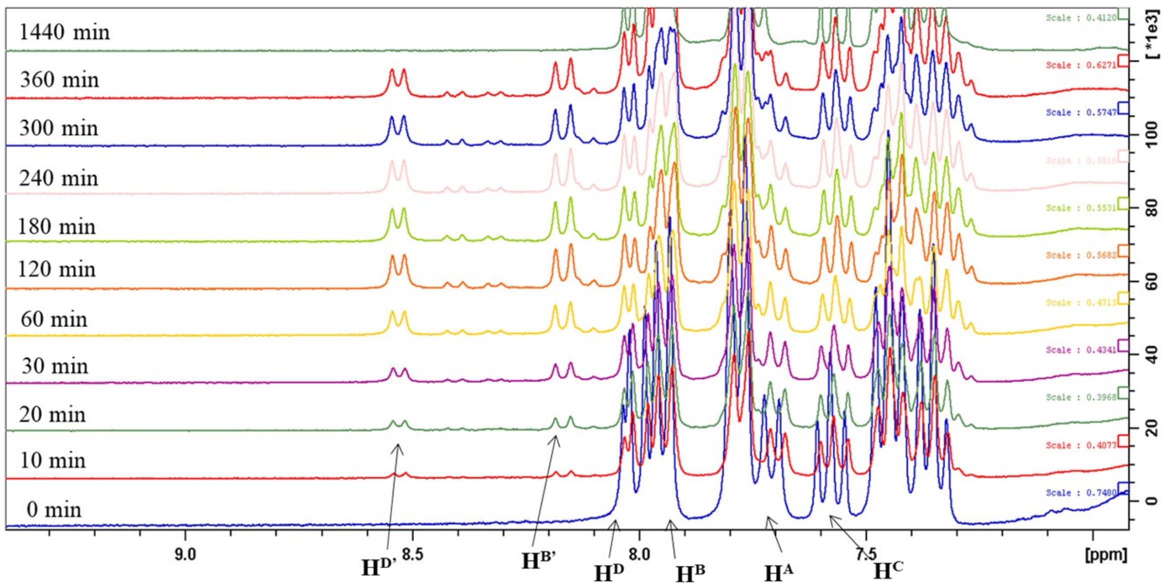

Figure 35. Reaction of Fmoc-Arg(Pbf)-OH and DIC/HOBt in DMF-d_7 as a function of time; ^{1}H NMR spectra, aromatic region, 250 MHz

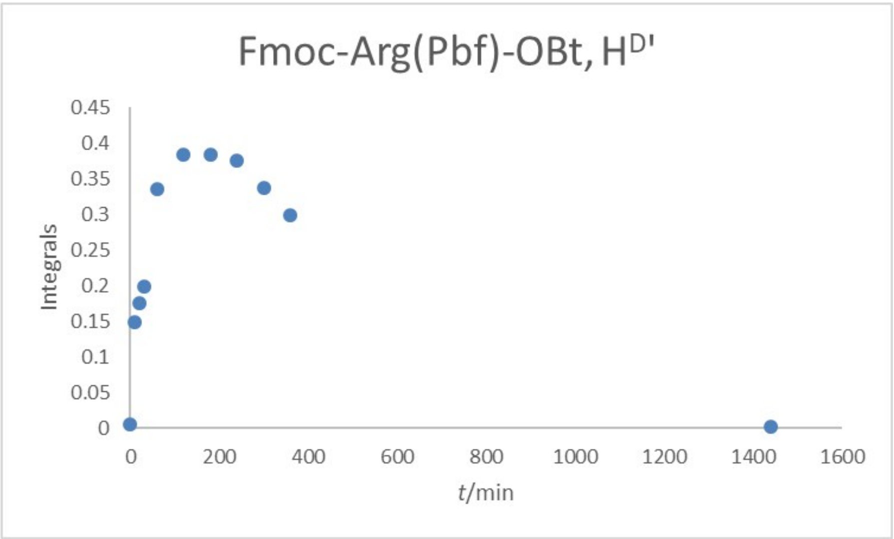

Figure 36. Integral-time diagram of Fmoc-Arg(Pbf)-OBt active ester, $H^{D'}$ signal at 8.52 ppm

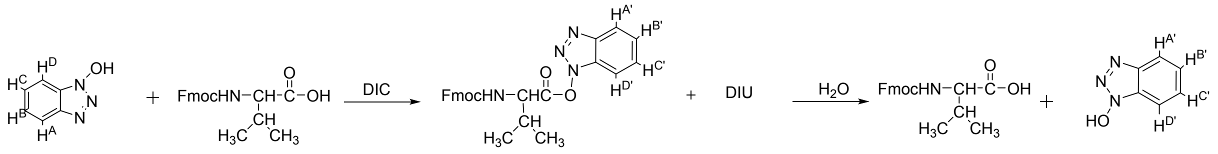

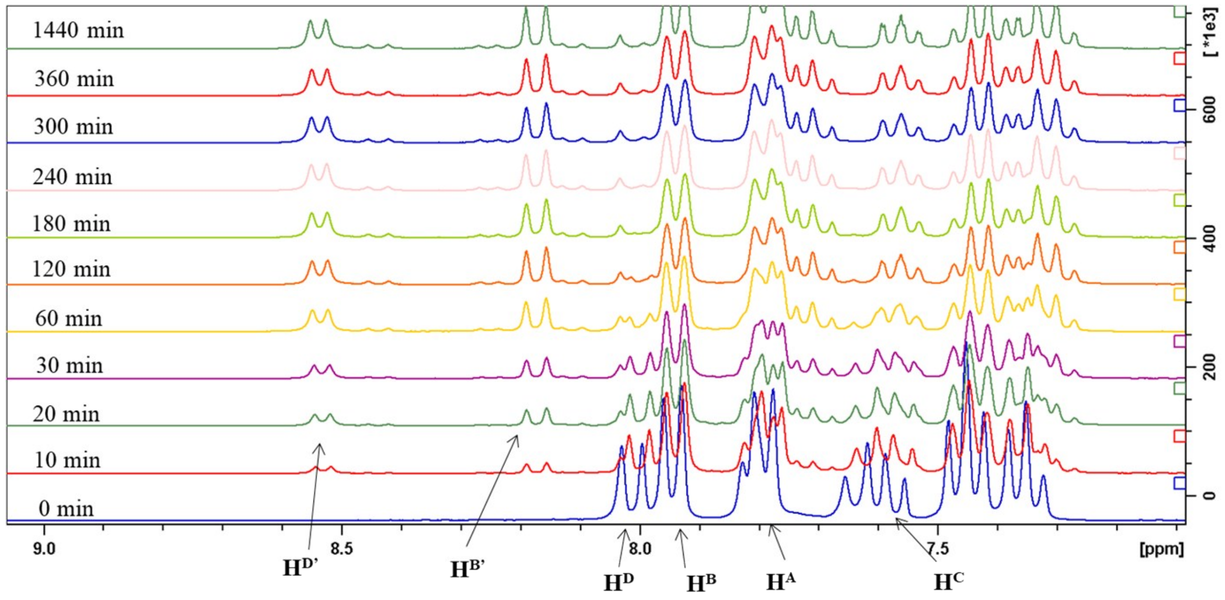

Figure 37. Reaction of Fmoc-Val-OH and DIC/HOBt in DMF-d_7 as a function of time; ^{1}H NMR spectra, aromatic region, 250 MHz

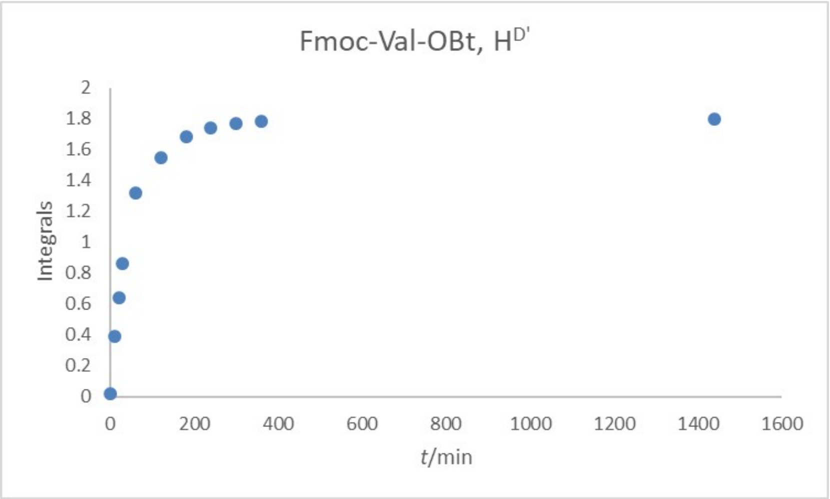

Figure 38. Integral-time diagram of Fmoc-Val-OBt active ester, $H^{D'}$ signal at 8.54 ppm

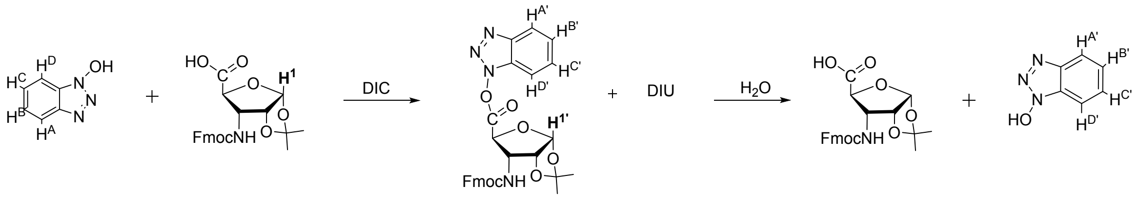

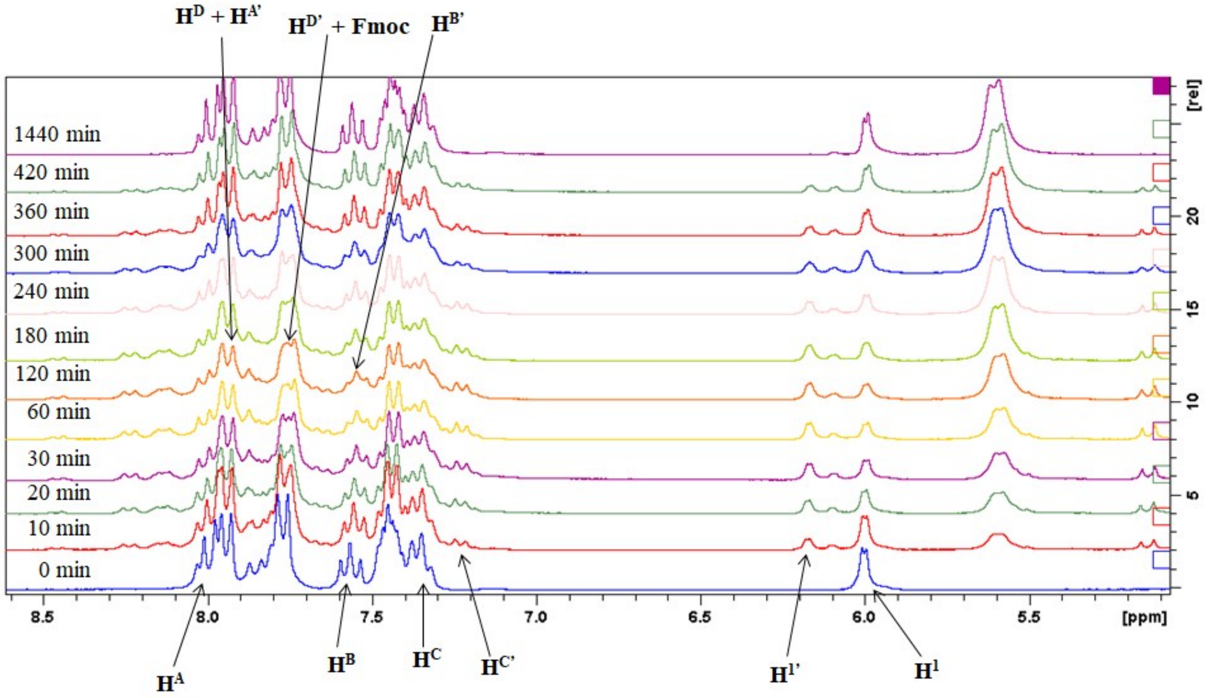

Figure 39. Reaction of Fmoc-RibAFU(ip)-OH and DIC/HOBt in DMF-d_7 as a function of time; ^{1}H NMR spectra, aromatic region, 250 MHz

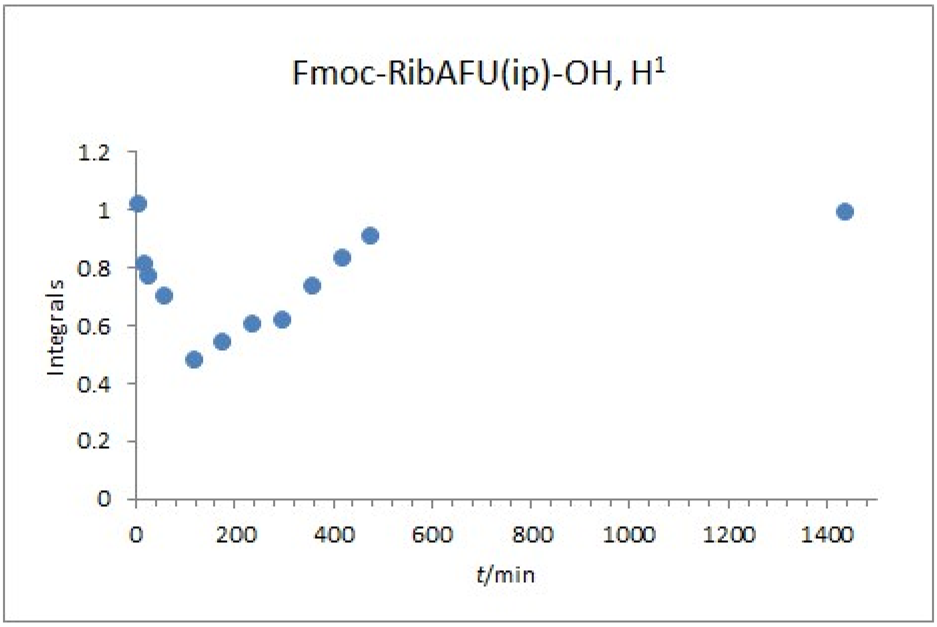

Figure 40. Integral-time diagram of Fmoc-RibAFU(ip)-OBt active ester, H$^{1'}$ signal at 6.19 ppm

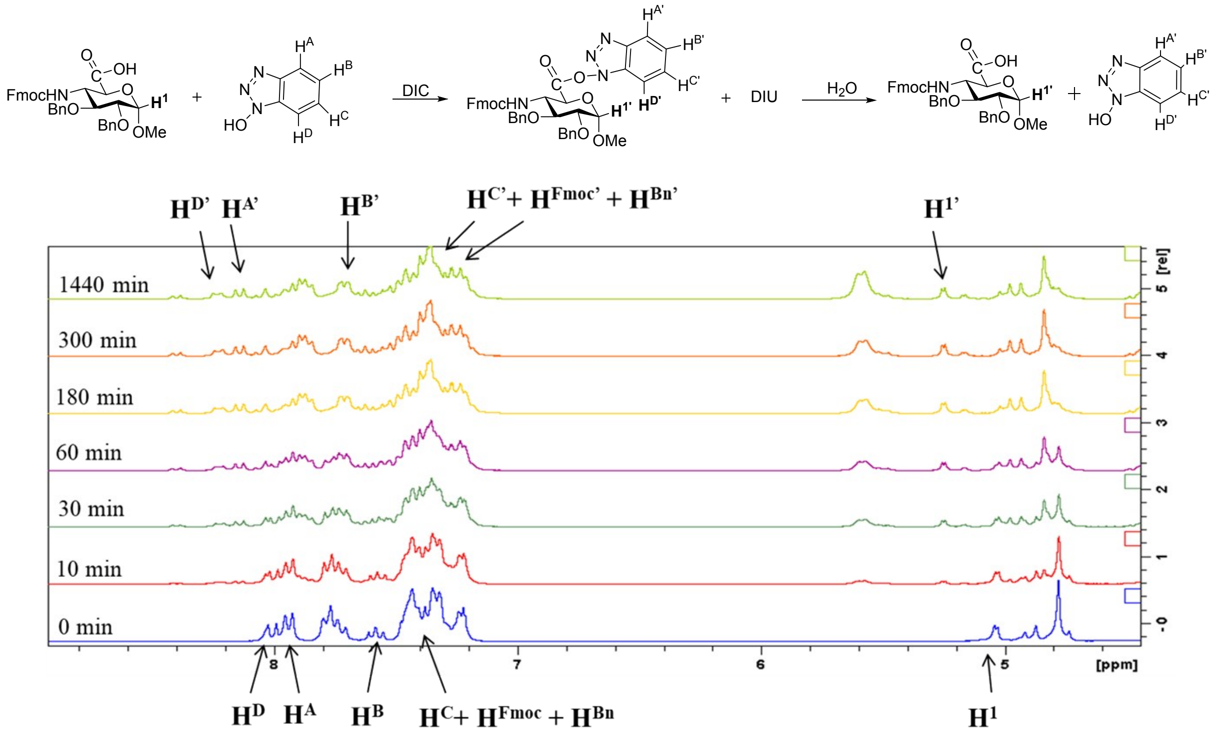

Figure 41. Reaction of Fmoc-GlcAPU(Me)-OH and DIC/HOBt in DMF-d_7 as a function of time; ^{1}H NMR spectra, aromatic region, 250 MHz

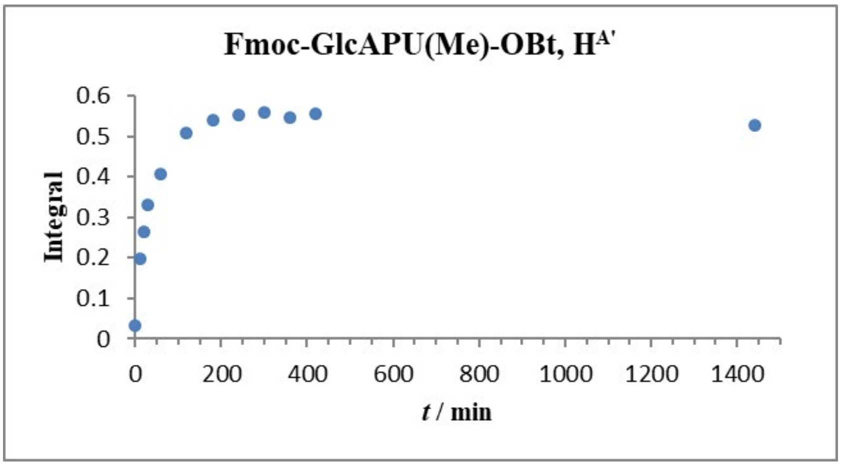

Figure 42. Integral-time diagram of Fmoc-GlcAPU(Me)-OBt active ester, H$^{1'}$ signal at 8.08 ppm

4. Kinetic analysis

Table 2. Parameter Estimation Summary: calculated parameters and their confidence intervals estimated using the COPASI 4.16 (Build 104)

**parameter**	**ndf**[a]	**t (0.975)**	$k_{(hydrolysis)}$					**$[AC]_{0,calc}$**					**$[H2O]_0$**				
**amino acis**			**Value**	Std. dev.	**int. hw**[b]	low	high	**Value**	Std. dev.	**int. hw**	low	high	**Value**	Std. dev.	**int. hw**	low	high
**Fmoc-Ile-OH**	3	4.177	**0.0031**	0.0715	**0.2987**	-0.2956	0.3018	**0.068**	0.0012	**0.0050**	0.063	0.073	**0.226**	5.142	**21.475**	-21.249	21.702
**Fmoc-Val-OH**	5	3.163	**0.0460**	0.0046	**0.0145**	0.0315	0.0605	**0.095**	0.0003	**0.0008**	0.094	0.096	**0.029**	0.001	**0.004**	0.024	0.033
**Fmoc-Arg(Pbf)-OH**	6	2.969	**0.1098**	0.2170	**0.6443**	-0.5345	0.7541	**0.021**	0.0007	**0.0022**	0.019	0.023	**0.184**	0.340	**1.011**	-0.827	1.195
**Fmoc-Ala-OH**	7	2.841	**0.0246**	0.0014	**0.0040**	0.0206	0.0286	**0.110**	0.0023	**0.0066**	0.103	0.116	cannot be estimated; fixed at 0.25 mM				
**Fmoc-Asn(Trt)-OH**	7	2.841	**0.0447**	3.87E-05	**0.0001**	0.0446	0.0448	**0.071**	0.0042	**0.0119**	0.060	0.083	**0.229**	0.156	**0.444**	-0.215	0.672
**Fmoc-Leu-OH**	4	3.495	**0.0504**	0.0068	**0.0237**	0.0267	0.0742	**0.069**	0.00081	**0.0028**	0.066	0.071	**0.093**	0.008	**0.029**	0.064	0.123
**Fmoc-GlcAPU(Me)-OH**	8	2.752	**0.0224**	0.0007	**0.0020**	0.0205	0.0244	**0.059**	0.00094	**0.0026**	0.056	0.061	cannot be estimated; fixed at 0.25 mM				
**Fmoc-Thr(^{t}Bu)-OH**	3	4.177	**0.0248**	0.0028	**0.0117**	0.0131	0.0365	**0.059**	0.00038	**0.0016**	0.057	0.061	**0.095**	0.00757	**0.032**	0.064	0.127
**Fmoc-βAla-OH**	2	6.205	**0.0073**	0.00066	**0.0041**	0.0032	0.0114	**0.136**	0.0026	**0.0162**	0.120	0.152	cannot be estimated; fixed at 0.25 mM				
**Fmoc-GalAPU(Me)-OH**	6	2.969	**0.0092**	0.00027	**0.0008**	0.0084	0.0100	**0.060**	0.0011	**0.0034**	0.056	0.063	cannot be estimated; fixed at 0.25 mM				
**Fmoc-Gly-OH**	8	2.752	**0.0449**	0.0097	**0.0266**	0.0183	0.0715	**0.102**	0.0013	**0.0036**	0.098	0.106	**0.1423**	0.020151	**0.055**	0.087	0.198

a) is the number of degrees of freedom of the associated Student distribution calculated as the number of data – the number of parameters.

b) always stands for half width of the 95% confidence interval

Some datasets (where there was a monotonous decay of the active ester concentration) used for parameter estimation contain the first 10/20 minutes measured values as well.

If the coupled active ester had an initial plateau, zero time of hydrolysis has been assigned to the last point on the plateau before concentration decay.

Parameters whose confidence interval is large and also contains zero are not determined significantly.

Table 3. Measured and estimated concentration of active esters ($[AC]_0$)

Amino acids	$[AC]_0$		conf. interval	
	**measured**	**estimate**	low	high
**Fmoc-Ile-OH**	**0.0724**	**0.0684**	**0.0634**	**0.0734**
**Fmoc-Val-OH**	**0.0950**	**0.0942**	**0.0941**	**0.0958**
**Fmoc-Arg(Pbf)-OH**	**0.0174**	**0.0238**	**0.0123**	**0.0353**
**Fmoc-Ala-OH**	**0.0998**	**0.1095**	**0.1030**	**0.1161**
**Fmoc-Asn(Trt)-OH**	**0.054**	**0.0714**	**0.0595**	**0.0832**
**Fmoc-Leu-OH**	**0.0688**	**0.069**	**0.066**	**0.071**
**Fmoc-GlcAPU(Me)-OH**	**0.051**	**0.059**	**0.056**	**0.061**
**Fmoc-Thr(^{t}Bu)-OH**	**0.059**	**0.059**	**0.0573**	**0.0605**
**Fmoc-βAla-OH**	**0.132**	**0.136**	**0.1198**	**0.1523**
**Fmoc-GalAPU(Me)-OH**	**0.0313**	**0.0597**	**0.0564**	**0.0631**
**Fmoc-Gly-OH**	**0.1061**	**0.1021**	**0.0985**	**0.1058**

ParameterEstimationResults.zip contains three files for each parameter estimation of different active ester hydrolysis kinetics as follows:

***.dat** files contain the input data of the parameter estimation
(Note that some of the data at the and are sometimes ignored.)

***.txt** files contain the output results of parameter estimation
(As produced by COPASI.)

***.tiff** files contain a diagram showing measured data and the fitted curve
(As produced by COPASI.)
